# Revolutionizing environmental cleanup: the evolution of MOFs as catalysts for pollution remediation

**DOI:** 10.1039/d4ra05642f

**Published:** 2024-11-20

**Authors:** Umme Farwa, Zeshan Ali Sandhu, Azwa Kiran, Muhammad Asam Raza, Sufyan Ashraf, Hamza Gulzarab, Muhammad Fiaz, Adnan Malik, Abdullah G. Al-Sehemi

**Affiliations:** a Department of Chemistry, Faculty of Science, University of Gujrat, Hafiz Hayat Campus Gujrat 50700 Pakistan asamgcu@yahoo.com; b Department of Chemistry, Faculty of Science, University of Engineering and Technology Lahore Lahore Pakistan; c Department of Chemistry, University of Texas at Austin USA; d Department of Physics and Chemistry, Faculty of Applied Science and Technology, University Tun Hussein Onn Malaysia Pagoh Campus Malaysia; e Central Labs, King Khalid University AlQura'a, P.O. Box 960 Abha Saudi Arabia

## Abstract

The global problem of ecological safety and public health necessitates, the development of new sustainable ideas for pollution remediation. In recent development, metal–organic frameworks (MOF) are the emerging technology with remarkable potential, which have been employed in environmental remediation. MOFs are networks that are created by the coordination of metals or polyanions with ligands and contain organic components that can be customized. The interesting features of MOFs are a large surface area, tuneable porosity, functional diversity, and high predictability of pollutant adsorption, catalysis, and degradation. It is a solid material that occupies a unique position in the war against environmental pollutants. One of the main benefits of MOFs is that they exhibit selective adsorption of a wide range of pollutants, including heavy metals, organics, greenhouse gases, water and soil. Only particles with the right combination of pore size and chemical composition will achieve this selectivity, derived from the high level of specificity. Besides, they possess high catalytic ability for the removal of pollutants by means of different methods such as photocatalysis, Fenton-like reactions, and oxidative degradation. By generating mobile active sites within the framework of MOFs, we can not only ensure high affinity for pollutants but also effective transformation of toxic chemicals into less harmful or even inert end products. However, the long-term stability of MOFs is becoming more important as eco-friendly parts are replaced with those that can be used repeatedly, and systems based on MOFs that can remove pollutants in more than one way are fabricated. MOFs can reduce waste production, energy consumption as compared to the other removal process. With its endless capacities, MOF technology brings a solution to the environmental cleansing problem, working as a flexible problem solver from one field to another. The investigation of MOF synthesis and principles will allow researchers to fully understand the potential of MOFs in environmental problem solving, making the world a better place for all of us.

## Abstract 2

Recently, metal–organic frameworks (MOFs) are emerging technology with remarkable potential, which have been employed in environmental remediation.

## Graphical abstract

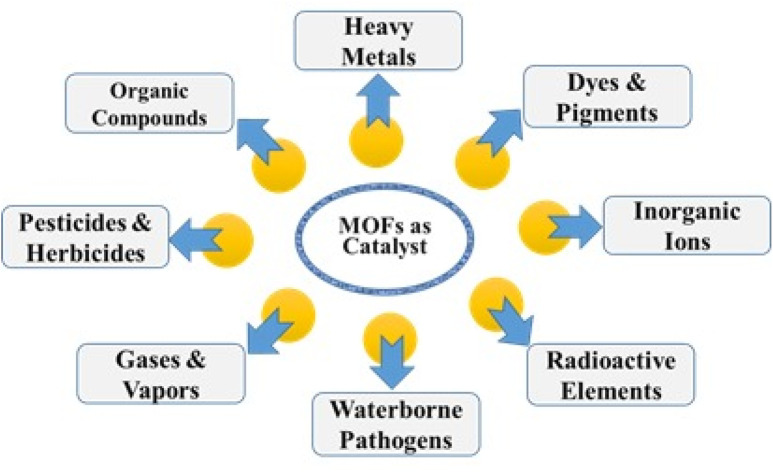

## 1 Introduction

The immense increase in environmental pollution carries a critical danger to ecosystems, human health, and the future of the earth's sustainability. Addressing this problem requires the application of new ideas and advanced technologies competent for removing and recycling pollutants.^1^ In the past few years, MOFs have been considered top environmental cleanup agents as they have demonstrably outstanding features and different applications in pollution remediation.^2^ MOFs, a group of porous and crystalline materials made up of metal cations attached to organic groups, have gained significant attention across diverse disciplines, such as separation and gas storage,^3^ catalysis,^4,5^ and sensing.^6,7^ With their precise tunability, large surface areas, and diverse functionalities, MOFs are the perfect materials for tailor-made solutions to marine and air pollutants.^8^ MOFs' development as a catalyst for pollutant removal definitely proves to be a vital achievement in the environmental chemistry and technology area.^9^ The advance of MOFs for separation and gas storage applications prompted researchers to discover their significant role in the mechanism of chemical reactions needed to clean polluted environments.^10^ Consequently, this finding sparked the phenomenon of using MOFs for gas adsorption, accumulation trapping, and detoxification of diverse water pollutants.^11^ One of the main benefits of using MOFs as catalysts for toxic substance removal can be seen in their extremely high surface-to-volume ratios and pores that provide space for the catalytic reactions to occur.^12^ MOF functionalization with catalytic moieties or metal nanoparticles enables researchers to direct and fine-tune the activity and selectivity of the catalytic processes targeted for pollution removal, which in turn leads to elevated efficiency and selectivity of the treatment.^13^

Additionally, the range of possibilities of using MOFs offers a chance to combine several functionalities into a single support structure, achieving multi-functional goals for environmental cleanup and causing synergistic effects.^14^ Multifunctional elements including photodegradation, sorption, catalysis, and membrane-based separation processes can be facilitated by the MOF system.^15^ The phenomenal rise of MOFs as the catalyst technology for pollution treatment and prevention has been powered by the astounding optical approaches, remarkable dynamic structural designs, and fascinating current synthesis procedures.^16^ It is difficult to compare the inventiveness of researchers with the transformation of all novel pathways for MOFs. Consequently, we were able to obtain chemical surface functionalization, metal–ligand bonding, and variable pore size by these methods. Moreover, MOFs have a higher sorption capacity and are controllably tailored to remove contaminants.^17^

As a result, state-of-the-art techniques such as SEM, XRD, and MOF spectroscopy provide insights into the structure–property relationship of MOFs as well as their catalytic processes, which are by no means employed in the process of removing pollution.^18^ For the purpose of improving the activity, stability, and recyclability of MOF catalysts for the removal of toxic wastes from industrial units, a highly successful design strategy based on MOFs has been used.^19^ In addition to catalysis, more sophisticated techniques such as photocatalysis and electrocatalysis are now available for environmental remediation.^20,21^ As photocatalysts, MOFs play vital and indispensable roles, breaking down organic pollutants in UV or solar radiation when they absorb the right wavelength. Thus, the effective solar reaction of these MOFs with photocatalytic capabilities provides a universal, energy-efficient, and self-cleaning technique that can be employed without any additional factory equipment, without the need for additional chemicals or external energy sources.^22^

**Fig. 1:**
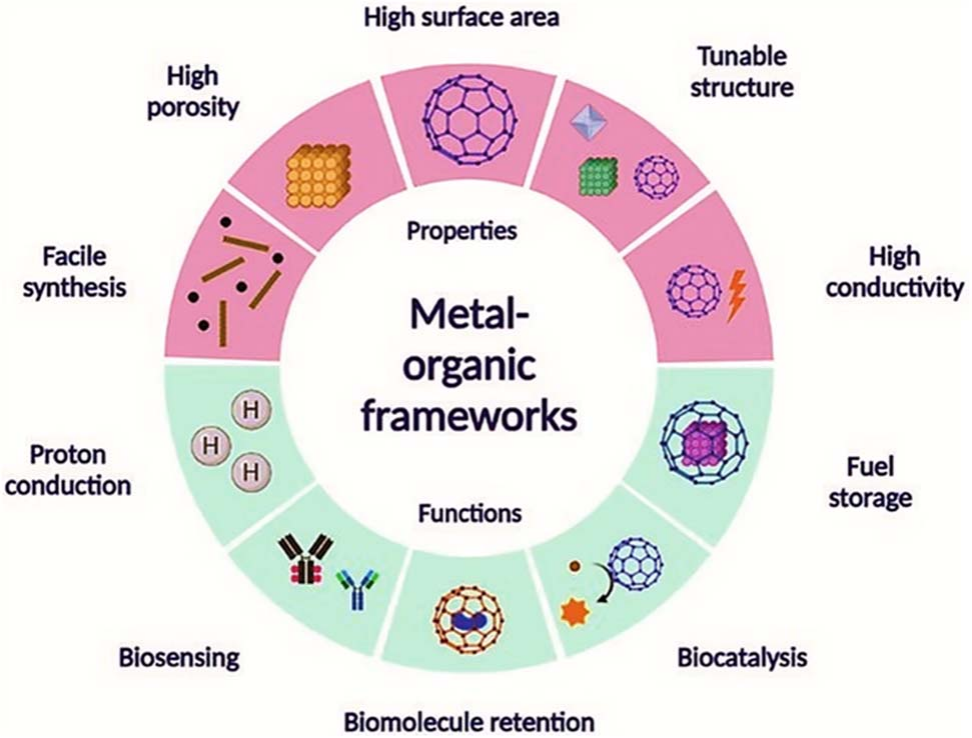
Properties and applications of metal organic frameworks. Reproduced with permission from ref. 27. Copyright (2022) The Royal Society of Chemistry.

MOF systems can be effectively employed in a range of electrochemical processes including the oxidation of contaminants, the evolution of hydrogen, and the reduction of oxygen due to their ability to alter their surface functionalities and electrical properties. For the decentralized and on-site disposal of different contaminants, primarily throughout the process of water treatment and wastewater management, this cutting-edge electrocatalytic approach may be promising.^23^ MOFs functionalized by entities with specific binding sites and recognition elements are highly selective for the target pollutants, allowing micro capture and concentration from complex matrices, making it easy to detect and quantify them using different analytical techniques.^24^ This function is very helpful for environmental monitoring and risk assessment, as it enables the identification of pollution sources and initiates relevant action to remedy the situation.^6^ Furthermore, the versatility of MOFs is not limited to their employment in themselves, as supports, or as immobilization matrices for other catalytic materials such as enzymes, metal nanoparticles, or molecular catalysts; they are therefore useful in water purification and environmental catalysis.^25^ Although steady progress has been achieved in this domain, there are still a number of bottlenecks to overcome to use MOFs maximally as catalysts in the purification of pollutants.^26^ These encompass the design of production methods that are scalable and cost-effective for fabricating MOFs with reliable properties and performance (Fig. 1).^28^ Additionally, the optimization of reaction conditions and chemically designing catalysts are to be considered in accordance with the efficiency, selectivity, and sustainability.^29^ Moreover, MOF catalysts' long-term ecological stability, recyclability, and overall environmental effects should be thoroughly assessed by proper life cycle assessment and environmental risk assessment methods.^30^ Addressing these hurdles will imply interdisciplinary contributions from material experts, chemists, engineers, and ecologists, whose focus will be on the integration of academic research with practical technologies and social application.

This work reviews the structure and application of MOFs as potential catalysts for pollutant treatment, discussing the important findings, differing factors, and future trends. The MOF's different applications in facilitating the removal of pollutants such as contaminants, metals, and chemicals through heterogeneous reactions in various matrices will be deliberated. Besides, the mechanisms that influence MOFs' catalytic activity considering different classes of pollutants as well as the strategies that are used to improve their performance and selectivity will be evaluated. Moreover, we will study the hybrids of MOFs with other materials or techniques, targeting the improvement of pollutant adsorption and driving sustainability in environmental cleanup processes. Through this review, the way in which MOFs impact the current standards for cleaning up industrial pollutants and the preferred direction of pollution control and remediation methods will be demonstrated. Through presenting current achievements, dealing with current issues, and listing upcoming prospects, our goal is to motivate more studies and technologies in the area constantly for future development.

## 2 Synthesis of MOFs

**Fig. 2:**
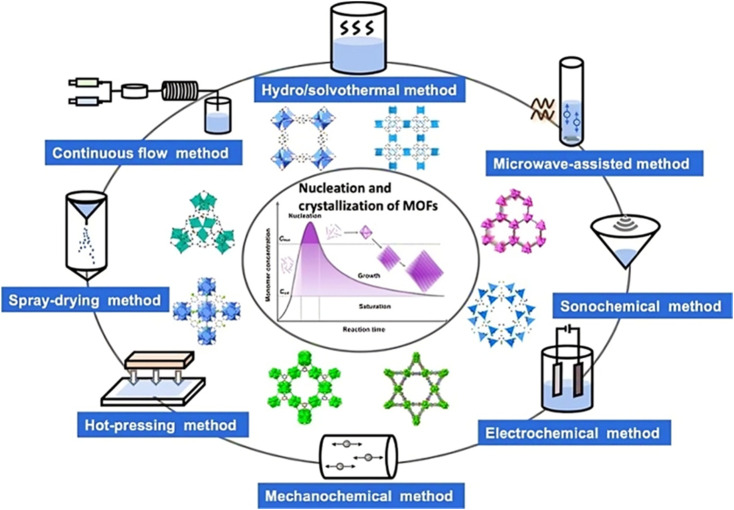
Representation of the synthetic scheme of metal organic frameworks. Reproduced with access provided by HEC.^34^

The synthesis of MOFs is influenced by several variables. These include reaction temperature and time, the chemical composition of metal ions, organic ligand solvent type, the size and structural properties of nodes, the presence of counter ions, and crystallization process facilitating nucleation.^31^ In general, solutions containing ligands and metal salts are combined to fabricate MOFs in the liquid phase. The stability constant, reactivity, redox potential, and solubility of the solvent are all influencing factors.^32^ In this method, the raw materials are dissolved in a mixture of solvents. Subsequently, the solvent gradually evaporates under controlled conditions, usually in an inert environment and at a specific temperature.^33^ This traditional approach for synthesizing Metal–Organic Frameworks (MOFs) does not rely on an external energy source.^33^Fig. 2 illustrates the synthetic scheme of MOFs.

A facile melt-diffusion approach is used to form hollow metal–organic framework (MOF) composites coated with polypyrrole, which have the potential to be employed in high-performance Li–S batteries.^35^ Due to their fascinating structural topologies, exceptional stability, endurance, and functionality, Fe-MOFs have attracted a lot of attention. Post synthesis changes are then introduced, and their prospective uses in catalysis, gas storage, and sensors are described.^36^ By addition of acetic acid, H_2_BDC, H_2_O, and ZrCl_4_ to a conical flask containing *N*,*N*′-dimethylformamide, the diffusion process is carried out to synthesize UiO-66. The mixtures are stirred until they form a clear solution.^37^

**Fig. 3:**
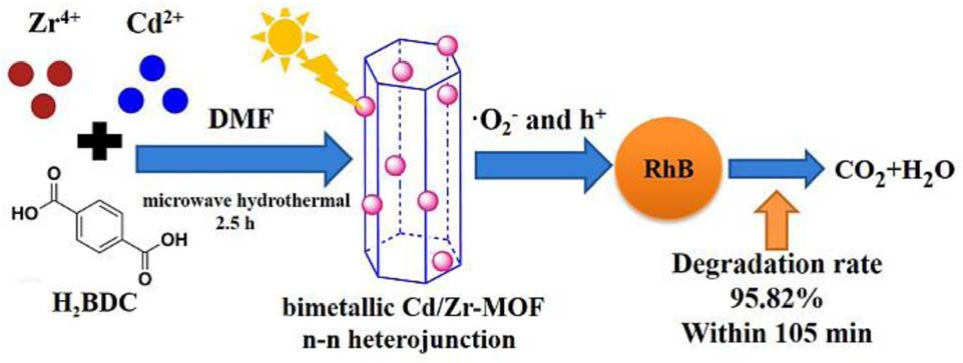
Synthetic and photocatalytic activity of synthesized Cd/Zr-MOF nanomaterials. Reproduced with access provided by Higher Education Commission (HEC).^41^

Using the hydrothermal synthesis process, crystalline materials are produced from water solutions at temperatures between 80 and 220 °C while autogenous pressure is applied. Steel autoclaves with PTFE liners that are tightly closed are used for this. The process can produce single crystals, depending on the material's solubility in boiling water at high vapor pressures.^38^ The process is known as the solvothermal technique when it is executed with solvents other than water. This technique fosters the development of high-quality crystals and is suitable for materials with vapor pressure close to their melting points.^39^ However, this method has drawbacks such as its lengthy operational time (potentially 3–4 days) and the difficulty of monitoring crystal growth. Another method, ion thermal synthesis, involves producing MOFs in the presence of ion-containing liquids without the use of organic solvents. This approach has led to several successful instances.^40^ A novel bimetallic metal–organic framework (Cd/Zr-MOF) was successfully synthesized by Cheng *et al.*, (2021) using a microwave hydrothermal technique, using Zr^4+^ and Cd^2+^ as metal ions and terephthalic acid (H_2_BDC) as the organic ligand. The influence of Cd/Zr molar ratio and reaction temperature on the Cd/Zr-MOF structure and its photocatalytic activity for removing Rhodamine B under simulated sunlight was studied.^41^ The synthetic mechanism and catalytic activity of Cd/Zr-MOF is depicted in Fig. 3.

**Fig. 4:**
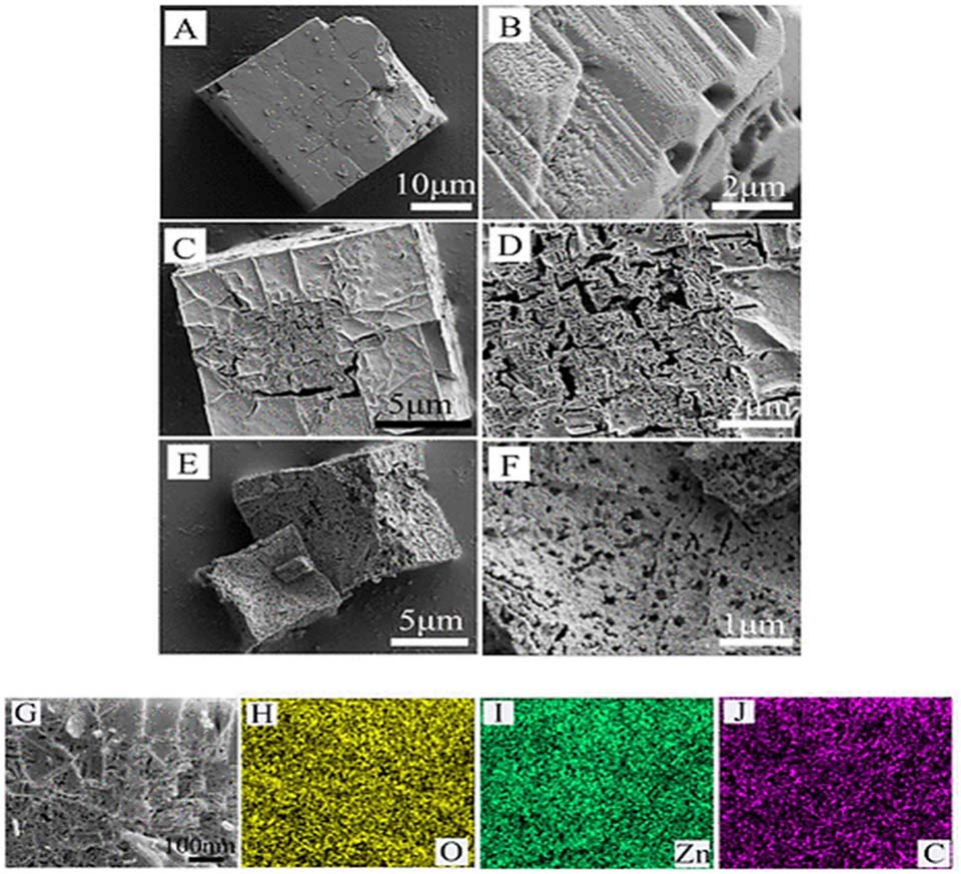
SEM images of M-MOF-5 (A and B), M-ZnO-500 analysis (C and D), and M-ZnO-550 (E and F). SEM image (G) and EDX elemental mapping (H–J) of M-ZnO-500. Reproduced with permission access provided by the University of Gujrat.^42^

Wang *et al.* (2020) prepared MOF-5 microwave hydrothermally. In a typical method, 2.149 g Zn(NO_3_)_2_·6H_2_O and 0.600 g H_2_BDC are mixed in 60 mL DMF. Sample SEM and elemental mapping images are shown in Fig. 4. As-synthesized M-MOF-5 has a well-defined, cubic structure with ∼25 μm side length. After calcination, the samples retain the cubic shape of M-MOF-5, but the surface becomes porous and rough, and the cube side length is shorter (M-ZnO-500 ∼15 μm and M-ZnO-550 ∼10 μm).^42^

**Fig. 5:**
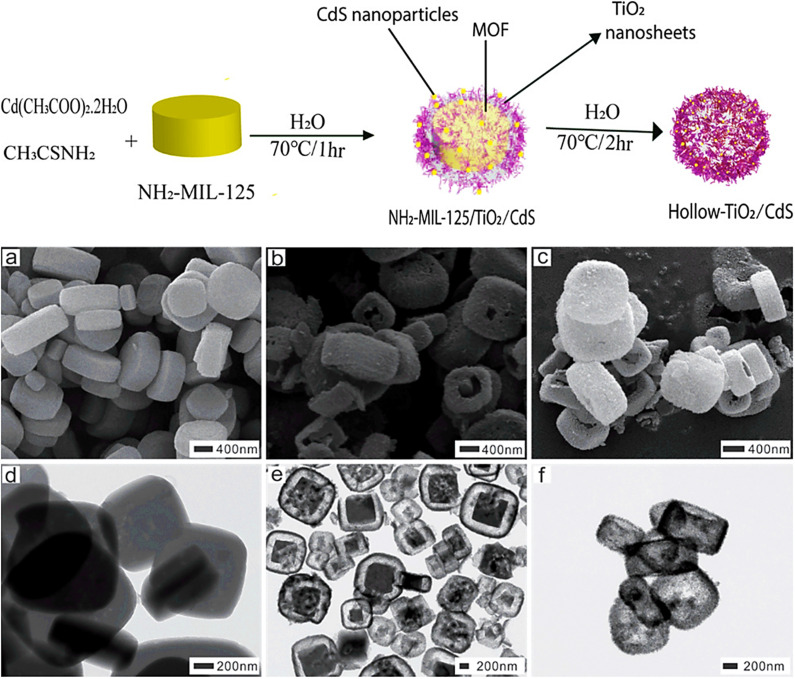
Synthesis of NH_2_-MIL-125/TiO_2_/CdS yolk–shell and hollow H-TiO_2_/CdS heterostructures. SEM (a–c) and TEM (d–f) images of NH_2_-MIL-125 MOF, NH_2_-MIL-125/TiO_2_/CdS yolk–shell, and H-TiO_2_/CdS. Reproduced with permission from ref. 48. Copyright (2018) American Chemical Society.

Tzitzios *et al.* (2017) synthesized IRMOF-1 in DMF using solvothermal reactions. Zinc nitrate and terephthalic acid were combined in dimethylformamide to construct crystalline, nanoporous MOFs. In addition to completely reversible H_2_ sorption behavior, activated IRMOF-1 demonstrated gravimetric H_2_ absorption.^43^ The solvothermal approach is used in autoclaves made of polypropylene to create the known MOF {Zn_4_O(BDC)_3_} (MOF-5 (I), which has terephthalate anions (BDC).^44^ A solvothermal process was used for the synthesis of Mn-MOF/GO and Cu-MOF/GO and their structural characterization was also performed. When coupled with GO, MOFs can increase corrosion resistance and suppress corrosion in a synergistic manner.^45^ Using a straightforward solvothermal technique, Ni-MOF thin films without binders were created on a stainless steel substrate.^46^ Using trimethylamine, Zn-MOF-74 nanorods with consistent diameters of about 200 nm were effectively produced. A solvothermal assistant strategy was used for MOFs, and researchers investigated the properties of TEA on the dimensions and configurations of nanoscale particles.^47^ Bibi *et al.* (2018) synthesized visible-light-active NH_2_-MIL-125/TiO_2_/CdS yolk–shell and hollow H-TiO_2_/CdS hybrid heterostructure MOFs using NH_2_-MIL-125 MOF as a metal precursor *via* a solvothermal technique. Fig. 5(a–c) displays the SEM images of pellet-like formations with an average particle size of 1 μm. The TEM images in Fig. 5(d–f) show that the heterostructure had a yolk (MOF) and shell (TiO_2_/CdS) with a void space, while the other H-TiO_2_/CdS has a hollow shape, confirming yolk–shell and hollow heterostructures.^48^

**Fig. 6:**
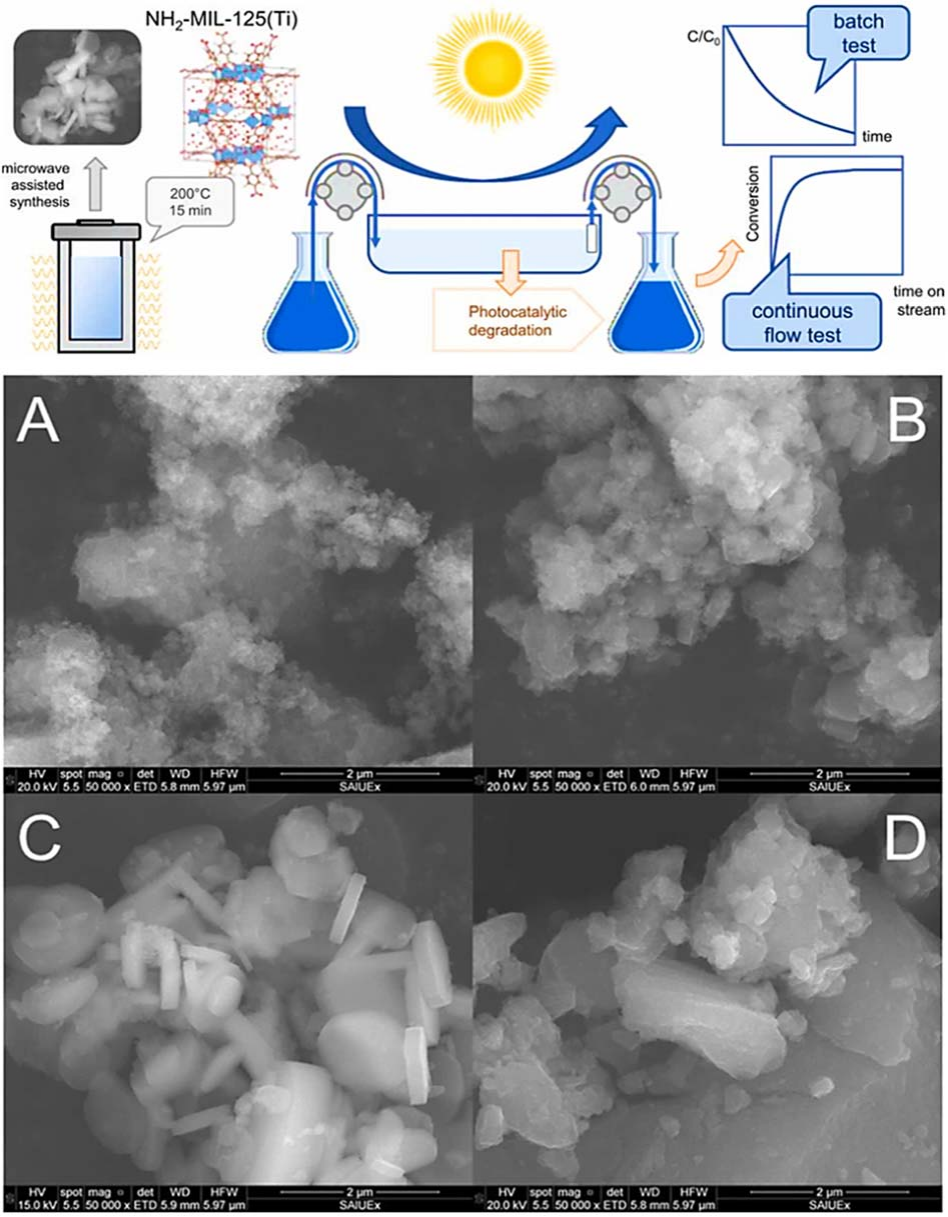
Schematic of the microwave-assisted synthesis of NH_2_-MIL-125(Ti) samples at different temperatures and periods, and SEM images of the samples: (A) 140 °C for 15 minutes; (B) 160 °C for 15 minutes; (C) 200 °C for 15 minutes; and (D) 200 °C for 4 hours. Reprinted with permission from ref. 55. Copyright (2021) Elsevier.

Microwave irradiation is a common technique in organic chemistry. Recently, inorganic nanomaterials such as zeolites and this approach have also been used to synthesize MOFs.^49^ This method depends on the interaction of materials having mobile electric charges with electromagnetic radiation.^50^ A few studies demonstrating the efficiency of microwave radiation in the synthesis of lanthanide–organic frameworks are now accessible. With the aid of microwaves, Vakili *et al.* (2018) synthesized MOFs based on zirconium. They could examine the yield and porosity by adjusting the temperature, reaction time, and amount of modulator. The reaction took 24 hours to complete using the solvothermal approach, but it was finished in 2–2.5 hours using a microwave.^51^ A microwave-irradiation synthesis is an effective method to synthesize well-shaped, octahedral Zr-based metal–organic frameworks.^52^ Metal–Organic Frameworks of iron(iii) amino terephthalate that show potential applications in industrial and social fields have also been designed.^53^ In contrast to their monometallic counterparts, bimetallic MOFs with two distinct inorganic metal nodes might be more effective CO_2_ adsorbents. For CO_2_ adsorption, various bimetallic NiCo-MOF-74s produced with a microwave-assisted technique were examined.^54^ Solis *et al.* (2021) synthesized NH_2_-MIL-125(Ti) MOFs *via* microwave-assisted synthesis under various temperature conditions ranging from 140 to 200 °C for 15 min to 4 h holding period, as shown in Fig. 6.

**Fig. 7:**
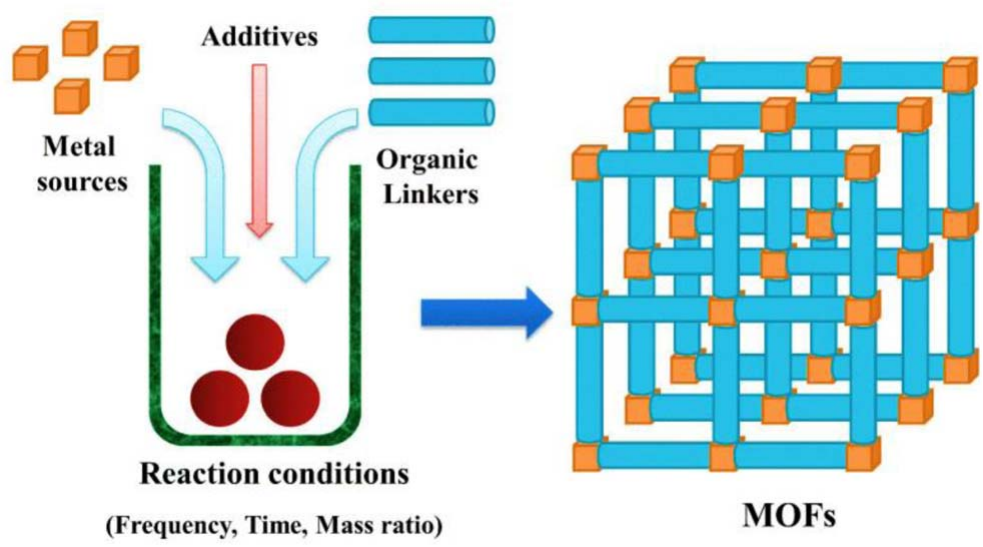
Synthesis of metal organic frameworks through ball milling process. Reprinted with permission from ref. 60. Copyright (2020) MDPI.

Moreover, mechanical chemistry offers an attractive alternative to the high temperature and pressure required for the solvo(hydro)thermal synthesis of MOFs. The main drawback of the approach is the challenge of isolating amorphous products, which are inappropriate for single-crystal X-ray structural analysis.^56^ Chen *et al.* (2017) mechanochemically created indium-based metal organic framework (InOF-1), which has been described as a promising substance for CO_2_ adsorption and separation.^57^ Mechanochemical processes generally use a small quantity of organic solvents or water to promote liquid-aided grinding and metal salts containing basic anions to deprotonate the conjugate acid of the organic linker, and both functions may be carried out by the liquid exogenous organic Hünig's base (*N*,*N*-diisopropylethylamine).^56^ The synthesis of copper-based MOF-505 has been successfully accomplished by a liquid-assisted mechanochemical technique. The type and quantity of the solvent that was supplied turned out to be key variables in the mechanochemical production of MOF-505.^58^ This method prevents excessive crystallization in the bulk phase by producing metal ions *in situ* close to the support surface. This reduction in undesired crystal accumulation is beneficial during membrane fabrication. Furthermore, compared to solvothermal synthesis, the lower temperatures in this technique appear to produce less obvious thermally induced cracking during the cooling phase.^59^Fig. 7 depicts the ball milling synthesis of MOFs.^60^

**Fig. 8:**
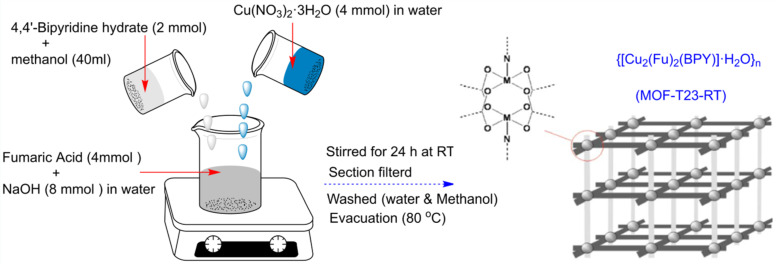
Synthesis of the pillared-layer MOF, {[Cu_2_(Fu)_2_(BPY)]·H_2_O}*n via* a chemical process.^65^ Reproduced with permission from ref. 65 Copyright (2022) RCS Advances.

This study's Mn-based diaminobenzenedicarboxylate MOF was synthesized using a chemical process.^61^ In a new electrochemical (EC) synthesis technique for the synthesis of large-area Cu-MOFs, a charge-induced molecule assembly was used to accomplish the surface reaction.^62^ The current work covers the electrodeposition approach used to produce 3D nucleated micro particles on glassy carbon electrodes (GCE) such as Cu-MOF(MPsLCu-MOF)-SWCNT composites.^63^ As an alternative to ultrasonic cleaning baths, laboratory ultrasonic horns provide ultra-sonication up to 100 times more intensely for sonochemical operations. The ultrasonic horn is immersed directly in the sample container while the response vessel is positioned in the ultrasonic cleaning bath. This distinction is the key factor distinguishing ultrasonic cleaning baths from ultrasonic horns.^64^ Habtemariam *et al.* (2022) developed {[Cu_2_(Fu)_2_(BPY)]·H_2_O}*n*, a pillared-layer MOF, at ambient temperature using water and methanol as the reaction solvent. With sodium salts of fumarate instead of native acids, the desired MOF may be easily produced in aqueous environments as illustrated in Fig. 8.^65^

**Fig. 9:**
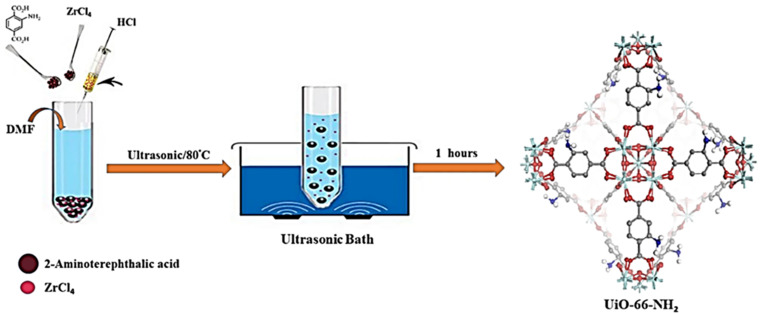
UiO-66-NH_2_ synthesized *via* a novel sonochemical method. Adopted with permission from ref. 71 Copyright (2023) Scientific Reports.

In this work, zirconyl chloride octahydrate and tetrakis(4-carboxyphenyl)porphyrin were combined to form Zr-based porphyrinic MOF-525 and MOF-545 in high purity and uniform size, respectively, using a sonochemical technique.^66^ Both solvothermal and sonochemical techniques have been used to fabricate a zinc-based Metal–Organic Framework (MOF) with adipic acid as an aliphatic ditopic linker.^67^ MIL-53(Fe), a metal–organic framework based on iron, was prepared *via* a quick and efficient simple sonochemical approach.^68^ The metal–organic framework (MOF) based on Zr-fumaric was successfully synthesized *via* sonochemistry, and the resulting MOF-based photocatalyst demonstrated 90% photocatalytic efficiency.^69^ The formation of Zn-MOF@chitosan and Cd-MOF@chitosan was achieved by the development of a sonochemical approach for the synthesis of 2-D Zn and Cd based MOF.^70^ Kazemi *et al.* (2023) adopted a novel method to synthesize UiO-66-NH_2_ within an hour, which was carried out with an ultrasonic equipment at 80 °C and atmospheric pressure for 60 min with constant concentrations of chemical reagents, as depicted in Fig. 9.^71^

Recent interest in two-dimensional conjugated metal–organic frameworks (2D c-MOFs) for possible applications in (opto-)electronics, chemiresistive sensing, and energy storage and conversion has grown due to their remarkable electrical conductivity, abundance of active sites, and intrinsic porosity architectures.^72^ Dodecanoic acid was used as a modulator to create titanium MOF nanoparticles with good control over size and colloidal stability and no effect on the framework's properties in order to directly build crystalline, porous thin films.^73^

## 3 Characterization of MOFs

**Fig. 10:**
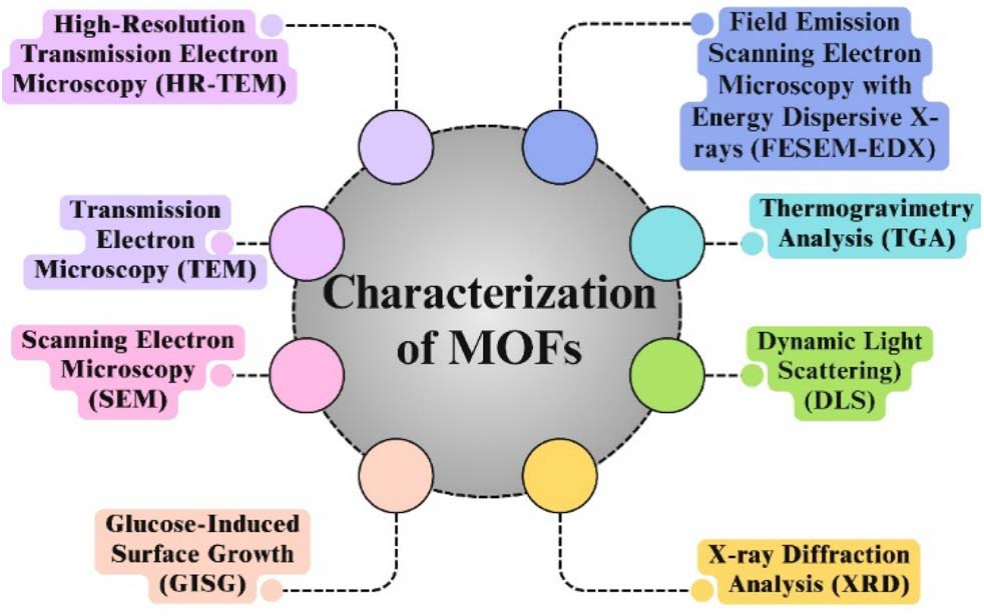
Different techniques used for the characterization of metal organic frameworks.

MOFs have potential applications in a various fields, including drug administration, sensing, ion exchange, gas storage, catalysis, molecular recognition, and separation. In order to understand how MOFs interact with other materials, it is crucial to use various characterization approaches.^74^ By TGA, DLS, XRD, and field emission scanning electron microscopy with energy-dispersive X-ray spectroscopy (FESEM-EDX) characterization techniques, the thermal stability, surface morphology, crystal structure, crystallinity, particle size distribution and chemical composition of the MOFs are all investigated.^75^ Metal ions and organic linkers combine to form crystalline nonporous metal–organic frameworks (MOFs), which significantly aid in the creation of porous materials with molecularly selective interfaces, unique physical characteristics, huge surface areas, and various functions.^76^ Usually, MOFs are characterized by using different techniques, as shown in Fig. 10.

**Fig. 11:**
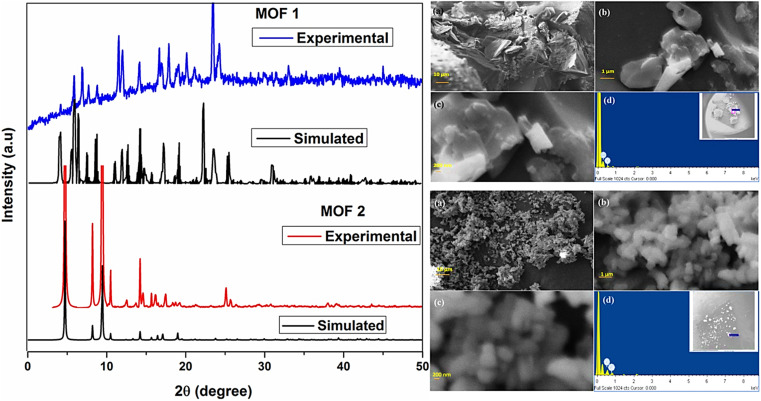
Left: XRD spectra of mixed-ligand [Co(BDC)(Phen)(H_2_O)](1) and [Co(BDC)(DABCO)](2) MOFs. Top right (a–d): SEM-EDX analysis of mixed-ligand [Co(BDC)(Phen)(H_2_O)](1) MOF. Bottom right (a–d): SEM-EDX analysis of mixed-ligand [Co(BDC)(DABCO)](2) MOF. Reprinted with permission from ref. 80. Copyright (2021) Springer.

To evaluate the crystallinity of the prepared materials, XRD analyses were performed using an X'Pert Pro MPD (PANalytical) diffractometer with CuK radiation at 30 mA and 40 kV. Using a 0.033° step, the diffraction patterns were acquired over the course of 12 minutes in the 2*θ* range of 5–80°. By the use of Grazing Incidence X-Ray Diffraction (GIXRD) analysis, the MOF layers formed on FeCrAl plates were evaluated for crystallinity. GIXRD analyses in the 5°–75° 2 range were performed using constant omega angles of 1° and 0.033°.^77^ To ascertain the MOFs' structural characteristics and crystallinity, PXRD is frequently used. By comparing the diffractogram of the synthesized MOF with an earlier one described in the literature, a simulated pattern produced by single-crystal X-ray and stored in a database, or by using computational modelling, the structural identification can be carried out.^78^ A Zn-BTC MOF has a 3D polymeric unit built by paddlewheel SBUs, according to a SXRD investigation.^79^ Chinthamreddy *et al.* (2021) used a solvothermal method to develop Mixed-ligand [Co(BDC)(Phen)(H_2_O)](1) and [Co(BDC)(DABCO)](2) MOFs at 150 °C. The molecular structure of MOF 1, determined by single-crystal XRD, is square planar with π⋯π contact between lateral 1,10-phenanthroline rings at 3.581 Å and 3.560 Å. The powder X-ray diffraction spectrum of MOF 2 suggests its crystalline character showing strong peaks below 10° (2*θ* values at 5.1, 8.1, 9.4, 11.3, 12.3, 16.2, and 18.7). SEM-EDX investigations of MOF 1 and 2 morphologies and elemental distribution were carried out. The SEM picture of MOF 1 displays a rough surface, pores, and heterogeneous crystals of different shapes and sizes, with an average particle thickness of 95 ± 12 μm. MOF 2 has needle-shaped morphology with 250–300 nm particle sizes. EDX spectrum testing confirmed MOF 1 and 2 contain cobalt at weight percentages of 21.04 and 22.23 and atomic percentages of 16.67 and 20.91, as shown in Fig. 11.^80^

One of the most popular methods for describing nanomaterials is scanning electron microscopy. The ethanol-based Zr-fum MOF NPs were synthesized which depicted spherical shape in SEM examinations.^81^ According to the SEM study, MPsLCu-MOF can develop on both bare GCE and SWCNTs with a size of 1 μm.^63^ In preparation, cubic MOF-5 has crystals and a porous nature.^82^ Co-based metal–organic frameworks of MOF films were visible in the top and cross-sectional views of the SEM on an ITO-coated glass substrate. According to Naeimi and Faghihian (2017), the MOF/Fe_3_O_4_/KNiFC SEM images are made up of scattered Fe_3_O_4_, MOF, and hetero-structured particles with an average particle size of 40–63 nm.^83^ Systems with several metals, linkers, or those containing guest species make good use of TEM. TEM imaging in NP@MOF systems can show the morphology and size distribution of the MOF crystals and embedded NPs. For electron tomography, the spatial relationship of observed components in 3D can also be ascertained if a series of pictures is obtained.^84^

**Fig. 12:**
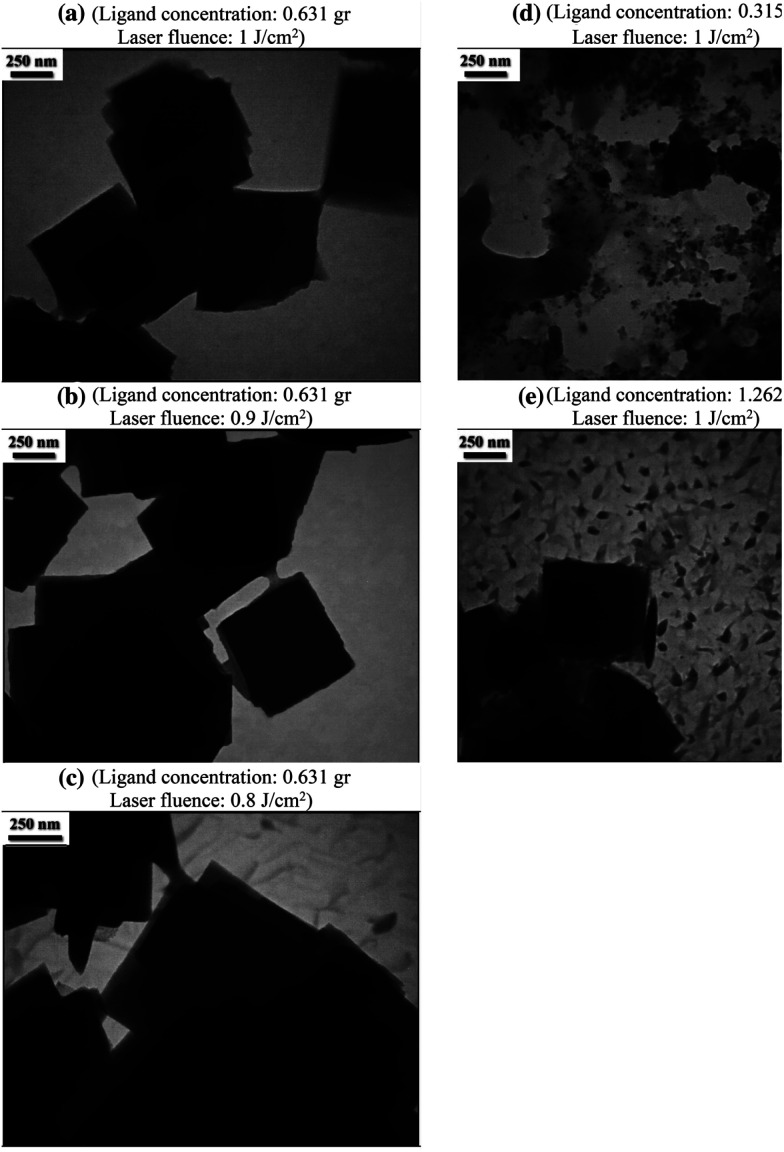
TEM pictures of MOF-5 synthesized using pulsed laser ablation (PLA) technique (250 nm scale). Reproduced with permission from ref. 87. Copyright (2021) Springer.

To verify the metal organic framework's structural integrity using a direct-detection electron-counting camera with a diamond structure, Zn_3_(HCOO)_6_ may obtain TEM images of MOF ZIF-8 at an ultralow dose of 4.1 Å electrons per square Å. The resulting image may resolve individual zinc atomic columns and organic linkers within the framework since it transmits structural information up to 2.1 Å.^85^ The HR-TEM micrograph for copper MOF reveals a broad dispersion of the metal nanocrystals as black dots uniformly dispersed in the solid matrix and crystalline solid's lattice structure was seen.^86^ Ataei *et al.* (2021) used the pulsed laser ablation (PLA) technique in a liquid environment as a physical bottom-up approach to fabricate the metal–organic framework MOF-5. Fig. 12 displays the TEM images of the nanostructures that were generated. The TEM images reveal that the produced MOF-5 possesses interior structures characterized by smooth surface cubic-shaped nanostructures of different sizes and concentrations.^87^

**Fig. 13:**
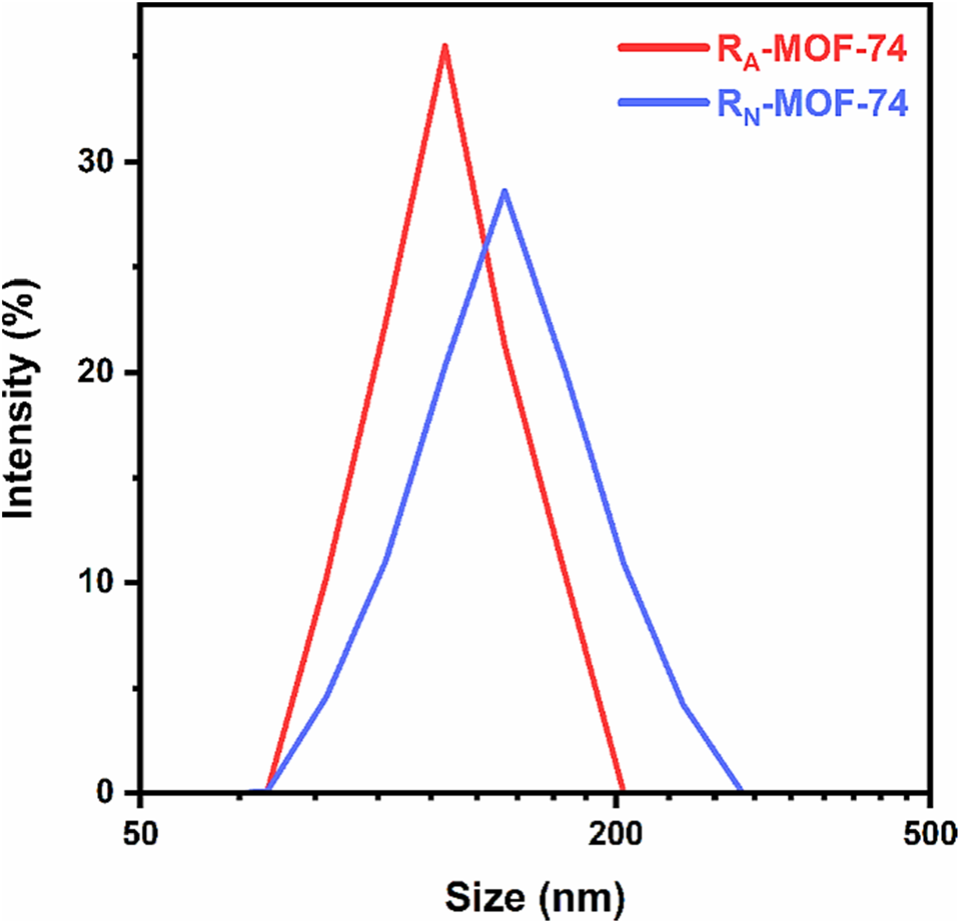
Dynamic light scattering (DLS) results of R_A_-MOF-74 and R_N_-MOF-74. Adopted with permission from ref. 88. Copyright (2024) Springer.

An experiment using dynamic light scattering (DLS) was carried out in water to determine the particle size. According to the DLS analysis, the Zn-BTC MOF has an average size of 415 nm, or almost a nanometer.^79^ Kazemi *et al.* (2024) adapted *in situ* polymerization to synthesise two polydopamine-coated Zn-MOF-74 nanocarriers, R_A_-MOF-74 and R_N_-MOF-74. DLS measurements were taken after 1 h ultrasonication of nanoparticles in ultrapure water. Sample R_A_-MOF-74 had a mean hydrodynamic radius of 119.2 nm and sample R_N_-MOF-74 139.7 nm. Sample R_A_-MOF-74 has a narrow, monodisperse distribution with a PDI of 0.066. In comparison, sample R_N_-MOF-74 had a moderate polydispersity index (PDI) of 0.16, as depicted in Fig. 13.^88^

**Fig. 14:**
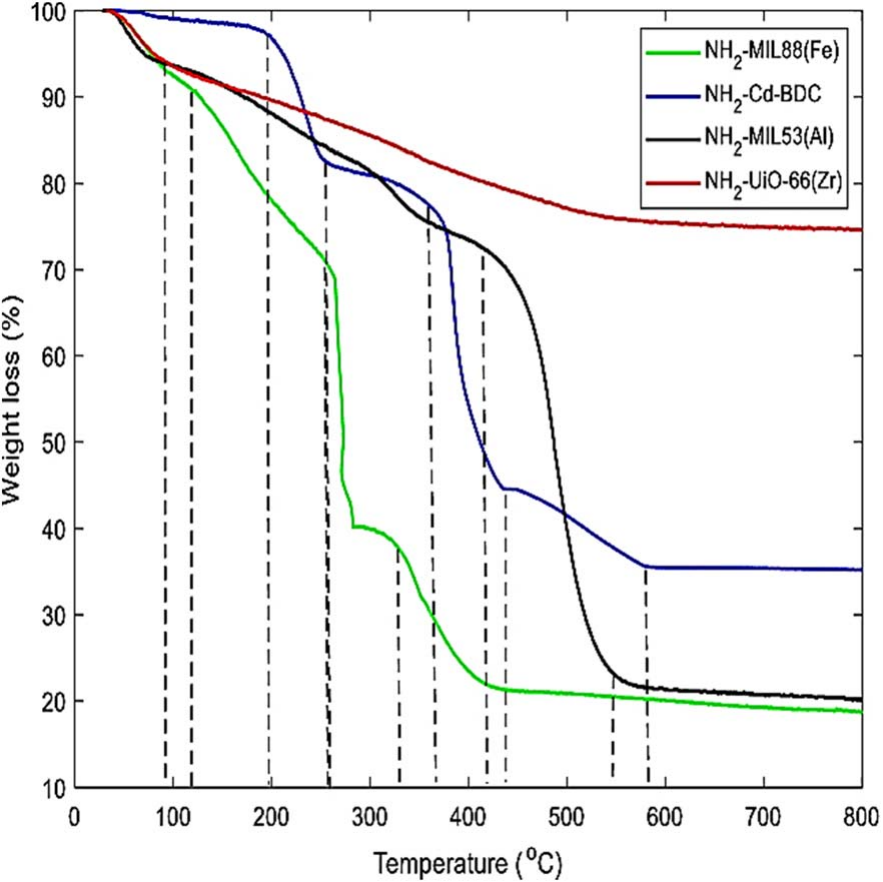
Thermogravimetric analysis (TGA) of four MOFs: NH_2_-UiO-66(Zr), NH_2_-MI(53), NH_2_-Cd-BDC, and NH_2_-MIL88(Fe). Reprinted with permission from ref. 90. Copyright (2022) Springer.

Thermogravimetric analysis (TGA) is an analytical method that uses weight changes during controlled heating to assess the volatile content and thermal stability of materials. TGA is particularly useful for polymers because it can track changes in weight loss as the temperature increases.^89^ Eltaher *et al.* (2022) synthesized four luminous MOFs: NH_2_-Cd-BDC, NH_2_-MIL53(Al), NH_2_-MIL88(Fe), and NH_2_-UiO-66(Zr) through a microwave-assisted method. Due to its high-temperature synthesis, the amorphous Zr-MOF is less susceptible to temperature increase than other MOFs, according to TGA. The other three MOFs exhibit two large reductions in TGA analysis, suggesting full rigidity loss as shown in Fig. 14.^90^

TGA tests monitor mass loss in a sample as it is heated steadily in a specific environment. For MOFs, this occurs in distinct phases. These phases often start with desolation at just around 150 °C. Following this, there is usually a plateau where the solvent-free evacuated MOF remains stable. Then, the framework begins to disintegrate, leading to a subsequent mass loss event. For example, heating a sample of HKUST-1 from room temperature to 125 °C reveals desolation.^91^Table 1 represents the synthetic approaches of MOFs.

**Table 1:** Synthetic approaches and characterization results of MOFs

MOFs	Methodology	Precursors	Conditions	Characterization	Results	References
Cu-MOF-1	Slow evaporation	(H_2_pdm = pyridine-2,6-dimethanol)	R.T	FT-IR, PXRD	An intense and distinct peak at 1384 cm^−1^ in the FTIR spectra of the indicates aromatic C–H stretching (*r*). Data from X-ray diffraction suggest that the product is highly pure	92
		Cu(NO_3_)_2_·3H_2_O, (bipy = 4,4′-bipyridine)				
Hf-MOF-808-DMF	Solvothermal	H_3_BTC (1,3,5-benzenetricarboxylic acid), HfCl_4,_ DMF/formic acid	Refluxed at 100 °C	SEM, PXRD, FT-IR	Both materials' FTIR spectra distinctly show the shift of the ∼1717 cm^−1^ signal, which is attributed to the carbonyl stretch. The MOF-808 structure has an identical crystal size of about 400 nm and is a pure crystalline phase	93
			Magnetic stirring 2 weeks			
NiCo-MOF	Solvothermal	Ni(NO_3_)_2_·6H_2_O, Co(NO_3_)_2_·6H_2_O, BPDC	Heated at 170 °C for 12 h, dried at 60 °C	SEM, XRD,FT-IR	The hierarchical structure of NiCo-MOF, which was synthesized using a hydrothermal process, is confirmed by the microspheres resembling hydrangeas that are formed from several 2D nanosheets	94
Cr-PTC-HIna	Solvothermal method	CrCl_3_·3H_2_O, Na_4_PTC, isonicotinic acid	Heated at 170 °C for 24 h	XRD, FT-IR, SEM	The crystal size of the Cr-PTC-HIna is 21 nm, absorption peaks are at wavenumbers 4000–400 cm^−1^ the observed peak at 545 cm^−1^ is correlated with Cr–O vibration, Cr-PTC-HIna that was 3D metal–organic frameworks with a cylindrical tube shape	95
Zr-MOFs	Solvothermal method	2-Amino-terephthalic acid, zirconium acetate	Heated at 150 °C for 14 h	FT-IR, XRD, SEM	The absorption maxima at 1581 and 1375 cm^−1^ are noteworthy. Furthermore, significant absorption peaks were seen between 1620–1550 and 1420–1300 cm^−1^, suggesting that carboxylic acids interacted with the metal salt and that Zr-MOFs had a better form and dispersion	96
MOF-74 (Ni)	Microwave-assisted synthesis	Ni(NO_3_)_2_·6H_2_O, (H_4_ dhtp = 2,5-dihydroxyterephthalic acid)	Heated at 125 and 140 °C for 60 min	FT-IR, XRD, SEM	Diffraction peaks for MW-140 are wider than those of the MW-125 sample, suggesting that as the nucleation temperature rose, smaller particles formed in MW-140	97
Ni-MOF	Microwave-assisted synthesis	Ni(NO_3_)_2_·6H_2_O, 2-methylimidazole (2-MI)	Heated at 150 °C for 50 min	FT-IR, XRD, SEM	The Ni-MOF's two distinct peaks, which are located at 11.0° and 22.1°, respectively, are associated with the (100) and (101) planes. The strong interaction between the C=N and C–H groups in 2-MI and the metal (Ni^2+^) ions was confirmed by the peaks at 2853 and 581 cm^−1^	98
ZIF-67	Microwave-assisted synthesis	2-Methylimidazole, Co(NO_3_)_2_·6H_2_O	Heated for 5 min	FT-IR, XRD, SEM	The samples remain in their rhombic dodecahedral shape. The SF samples are typically 500 nm in size. Good alignment of the (110) direction was observed from the greatest peak at 2 theta value of 7.3°, representing the (110) plane of ZIF-67 crystals	99
Zn-MOF-2	Microwave-assisted synthesis	Zn(NO_3_)_2_·6H_2_O, d benzene-1, 4-dicarboxylate acid	Microwave irradiation (10 W for 9 min)	FT-IR, XRD, SEM	Zn-MOF-2 SEM pictures were obtained at various magnifications, including 1 μm and 200 nm. Zn-MOF-2 was observed as a variety of sized, flakes-like particles. The region of the C–O stretching frequency corresponds to a strong band that is centered between 1345 and 1341 cm^−1^	100
Zinc-based pillar-layer MOF 1	Mechanochemical synthesis	Zn (CH_3_COO)_2_·2H_2_O, (5-aip, aminoisophthalic acid), (bpy, 4,4′- bipyridine)	40 Hz, 1 to 5 min	PXRD, SEM	With good crystallinity and great purity, the distinctive peaks of MOF-1 emerge at 2*θ* of 7.5°, 11.5°, 13.9°, 15.9°, and 18.3°. The SEM pictures clearly show that MOF-1 crystals have a lamellate shape	101
MOF-505	Mechanochemical synthesis	Cu(OAc)_2_·H_2_O, H_4_bptc	40 Hz, 20,40,60,80 100 min	PXRD, SEM	Apart from a little variation in peak intensity, PXRD signals revealed the absence of Cu(OAc)_2_·H_2_O. The mechanochemically synthesized MOF-505-K exhibited a semiregular cubic shape	58
MOF-5	Mechanochemical synthesis	Zn(OAc)_2_·2H_2_O, H_2_BDC	900, 1000, 1100 rpm, 30, 60, 90 min	PXRD, SEM	The PXRD patterns of the MOF-5 samples are depicted in the SEM image, which also shows a well-defined cubic structure and numerous tiny particles surrounding the MOF-5 crystals. The typical peaks at 6.8°, 9.7°, 13.7°, and 15.4° were present in all the samples	102
Cu_2_(BDC)_2_(DABCO)	Mechanochemical synthesis	Cu(OAc)_2_·H_2_O, H_2_BDC, DABCO	28 Hz, 120 min	FT-IR, XRD, SEM	A limited particle size distribution was evident in the SEM pictures of 8, 12, 16, 18, and 21 at 2 theta, where sharp peaks were visible. The majority of the particles' sizes fell between 40 and 60 nm, confirming the nano-cubic shape	103
MIL-53(Fe)	Sonochemical synthesis	Terephthalic acid, iron(iii) chloride hexahydrate	70 °C, 120 min	FT-IR, XRD, SEM	Strong diffraction peaks (200), (110), and (111) with high intensities were visible in the MIL-53(Fe) XRD pattern, suggesting the sample's high crystallinity. The infrared bands at 3450, 2940, 1597, 1393, and 540 cm^−1^ are associated with the aromatic C–H stretching vibration, the O–H stretching vibration, and the asymmetric and symmetric stretching vibration	104
MOF-525	Sonochemical	Zirconyl chloride octahydrate, benzoic acid, TCPP	3 h under different power conditions (30–60%)	SEM, XRD	Homogeneous cubic crystals, with MOF-525 represented by the XRD peak at 2*θ* = 4.8	66
[Zn_4_(oba)_3_(DMF)_2_ ] or TMU-39	Sonochemical	Zn(NO_3_)_2_·6H_2_O, H_2_oba	30 and 60 min, 12 W	SEM, FT-IR	The symmetric (COO) and asymmetric (COO) vibrations of the carboxylate groups are around 1400 cm^−1^ and 1600 cm^−1^, respectively	105
Cu(BDC)(DMF)	Surfactant-assisted synthesis	Cu(NO_3_)_2_, CTAB, BDC	5 h, 100 °C	FTIR	The C=O stretching mode of the free carboxylic acid groups is responsible for the band observed at 1670 cm^−1^. The coordinated carboxylic acid groups' symmetric and asymmetric stretching modes are responsible for the sharp bands located at 1394 and 1566 cm^−1^, respectively	106

## 4 Application of MOFs

### 4.1 MOFs as catalysts for the removal of heavy metals

**Fig. 15:**
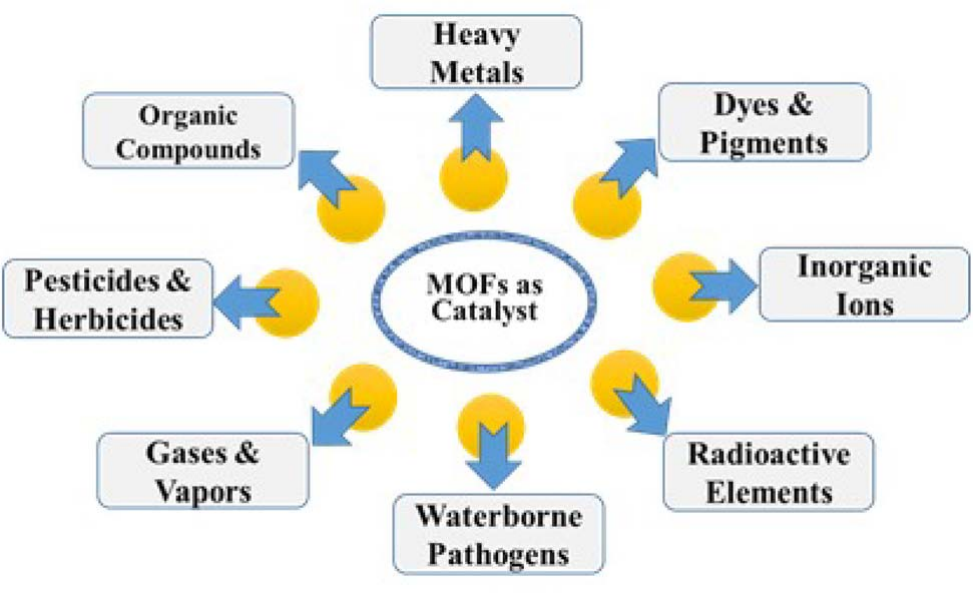
Representation of MOF performance as a catalyst for the removal of various pollutants.

A nano-sized (less than 100 nm), water-stable metal organic framework having zirconium metal with SO_3_H functionality, named UiO-66-SO_3_H, was used for effective lead(ii) adsorption from waste effluents. The adsorption capacity and removal rate achieved using this notable MOF were 176.6 mg g^−1^ and 88% respectively.^107^ Rapid and effective ultrasonic-assisted lead(ii) removal from wastewater was achieved using Cu-BTC and Zn-BTC MOFs. The highest adsorption capacities of these MOFs were 333 mg g^−1^ and 312 mg g^−1^, respectively, in less than 30 minutes, indicating that these compounds have greater adsorption capacities for the removal of Pb^2+^.^108^ Fe_3_O_4_^−^cysteine/NH_2_-MIL-53(Al), a magnetic MOF composite having a specific surface area of 322 m^2^ g^−1^, was used for the elimination of lead from wastewater. Because of its higher adsorption rate, *i.e.* 361.53 mg g^−1^, this MOF composite can be applied for lead removal on an industrial scale.^109^ A novel Ni-MOF with the chemical formula [Ni_2_F_2_(4,4′-bipy)_2_(H_2_O)_2_](VO_3_)_2_·8H_2_O was reported for showing the maximum uptake of lead(ii) up to 2400 mg g^−1^ from drinking water.^110^ A Cd-TPA-MOF (MOF-2) is used as a potential adsorbent for lead removal from wastewater. It showed the exceptionally high removal rate, *i.e.* only by using 1 g adsorbent dose, the removal rate is 99.9%. The adsorption isotherm follows the Langmuir model showing 434 mg g^−1^ adsorption rate.^111^ Cu_3_ (BTC)_2_-SO_3_H is a MOF with a sulfonic acid functionality, providing numerous binding spots and flexible coordination patterns for Cadmium and highly useful for Cd(ii) removal showing an adsorption capacity of 88.7 mg g^−1^.^112^ TMU-16-NH_2_ MOF was examined for the elimination of Cd(ii) from aqueous solutions. At an exposure time of 30 min and 6.0 pH, the percentage removal was 98.91 with an adsorption capacity of 126.6 mg g^−1^.^113^ A 3D CaFu MOF was used to remove hazardous Cd(ii) from an aquatic system *via* adsorption. The particle size of CaFu MOF is 30 nm with a truncated octahedron shape and good uniformity. The adsorption capacity achieved using this MOF was 781.2 mg g^−1^, and the percentage removal was 98.5% in 5 h.^114^ The *n*Fe_3_O_4_@MIL-88A (Fe)/APTMS MOF composite was synthesized by anchoring the amino group from APTMS onto the pore structure of iron MOFs. The particle size and BET surface area of this composite are 10–12 nm and 62.21 m^2^ g^−1^, respectively. It showed the maximum adsorption capacity of 755.8 mg g^−1^ at a concentration of 0.3 mol L^−1^.^115^ Fe_3_O_4_@UiO-66-NH_2_ and Fe_3_O_4_@ZIF-8 are the MOFs that are magnetic in nature and are highly selective in toxic Cd(ii) removal. Both have BET surface areas of 287 m^2^ g^−1^ and 160 m^2^ g^−1^. Fe_3_O_4_@UiO-66-NH_2_ showed a higher adsorption capacity for Cd(ii), *i.e.* 714 mg g^−1^ than Fe_3_O_4_@ZIF-8 having an adsorption capacity of 370 mg g^−1^_._^116^ The use MOF performance as a catalyst for the removal of various pollutants is depicted in Fig. 15.

The adsorption behavior of newly synthesized ZIF-67@GO (zeolitic imidazolate framework with graphene oxide) on Hg^2+^ has been studied through atomic adsorption spectrophotometry. The composite seemed to be highly efficient as it removed Hg^2+^ up to 91.1%.^117^ A thiol-modified uranium oxide MOF (UiO-66-SH) displayed high potential in removing Hg(ii) from wastewater by showing an adsorption capacity of 785 mg g^−1^ at a pH of 4. The MOF crystals have octahedral geometry and 250 nm particle size.^118^ PCN-221, a Zr-based MOF synthesized using H_2_TCPP (5,10,15,20-tetrakis(4-carboxyphenyl)porphyrin), was employed for Hg(ii) removal. The porphyrin ligand containing nitrogen functionalities contributed a lot in Hg(ii) adsorption showing 233 mg g^−1^ of adsorption capacity.^119^ A novel Zr-TDA MOF having thiodiacetic acid functionality with 74.37 m^2^ g^−1^ specific surface area and 10.03 nm pore size showed a high adsorption capacity of 605.5 mg g^−1^ for Hg(ii).^120^ An efficient MOF-808-SH was manufactured by fusing thioglycolic acid onto MOF-808 for the removal of Hg(ii). The particle size of prepared MOF is 500 nm with octahedral morphology. It showed an exceptionally quick adsorption kinetics (*C*_0_ = 10 ppm, percentage removal greater than 99 in 10 s) and an excellent uptake of Hg(ii), *i.e.* 977 mg g^−1^.^121^ Arsenic is a highly harmful heavy metal usually arising from fossil fuels, pesticides, smelting and mining. A high level of arsenic presents a significant danger to both human well-being and the environment. The Environmental Protection Agency in the United States mandates that the concentration of arsenic in drinking water must not exceed 10 ppb.^122^ Therefore, for the elimination of As-III (arsenite) and As-V (arsenate) from aqueous systems, a highly porous zeolitic imidazolate framework (ZIF-8) was employed. The As-III and As-V uptake shown by ZIF-8 at pH 8.6 was 2.02 and 1.42 mmol g^−1^ in a homogeneous system.^123^ In another study, cubic ZIF-8 and ZIF-8-ED MOFs (ED = ethylenediamine) with BET surface areas of 910 and 850 m^2^ g^−1^ respectively have shown arsenic uptake in the range of 72 to 83.5 mg g^−1^ at pH 7.^124^ An octahedral iron-based MOF, NH_2_-MIL-88(Fe), appeared to be capable of efficiently lowering minute concentrations of As(v) to levels below the acceptable water consumption standards. The adsorption capability of NH_2_-MIL-88(Fe) within an hour was 125 mg g^−1^.^125^ Another iron MOF, MIL-88B(Fe), having non-uniform needle-like crystals with 200 to 300 nm average length, was used for arsenate removal from drinking water. Even at low adsorbent doses, the adsorption capacity was maximum, *i.e.* 156.7 mg g^−1^, and at 6.4 μg L^−1^ adsorbate concentration, the adsorption capacity was 32.3 mg g^−1^, which meets the acceptable arsenic level for drinking water.^126^ UiO-66-36-TFA, a Zr based MOF with TFA as a regulator having 1690 m^2^ g^−1^ BET surface area, was employed for arsenic removal and has shown an arsenic uptake of 200 mg g^−1^ at pH 7. This high uptake is due to the unrestricted sites in Lewis acid which are formed due to the lost linker defects in MOF agglomerates.^127^

**Fig. 16:**
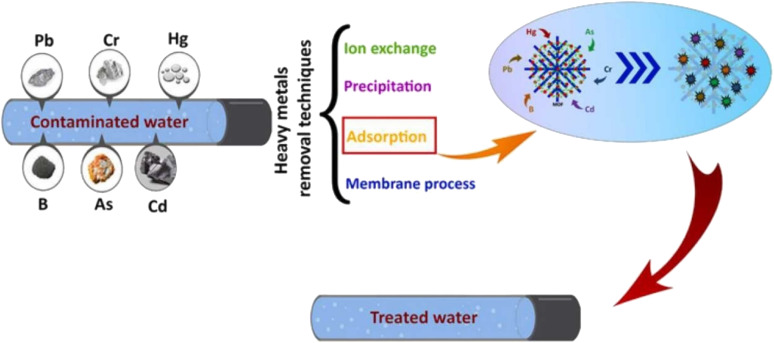
Mechanistic representation of MOFs as photocatalysts for the degradation of heavy metals. Reproduced with permission ©copyright ref. 133.

ZIF-8 (Zn-based zeolitic imidazolate framework) was investigated for its adsorptive behavior towards the removal of Cr(vi) pollutants from aqueous solutions. Within a contact time of 1 hour, the removal rate shown by 2 g of ZIF-8 MOF for 2.5 mg L^−1^ Cr(vi) solution was almost 70%. The adsorption of Cr(vi) anions was facilitated by the electrostatic interactions with positively charged ZIF-8.^128^ Ag-triazolato MOF with formula {[Ag_8_(tz)_6_](NO_3_)_2_·_6_H_2_O}*n* where tz = 3,5-diphenyl-1,2,4-triazolate; 1-NO_3_, has been studied for its anion exchange behavior. The crystals of Ag-MOF have a needle-like shape with 1–2 μm length. 1-NO_3_ exhibits robust, effective, and reversible adsorption of chromate ions (by anion-exchange) from the solution of Cr(vi) and the maximum adsorption capacity was 37 mg g^−1^ at 30 °C, representing it as a favorable MOF for Cr(vi) elimination.^129^ Fe_3_O_4_-ethylenediamine/MIL-101(Fe) MOF, magnetic in nature with 300 nm particle size, was employed for the pre-concentration of minute quantities of Cr(iii) ions, followed by their analysis by flame spectrophotometry. This magnetic MOF has shown a maximum adsorption capacity of 173 mg g^−1^ for Cr(iii) ions.^130^ A cationic, microporous Cd-MOF, [Cd (tipo)(HCOO)(H_2_O)]·NO_3_·DMF, was investigated for the removal of Cr(vi) ions from contaminated water. The addition of 10 mg of Cd-MOF into 15 mL of 50 ppm potassium dichromate solution results in 56% decrease in chromate ion (Cr_2_O_7_^2−^) concentration within 1 minute, and after 30 minutes, the removal of chromate ions increases to 91% with maximum Cr_2_O_7_^2−^ ion uptake of 228 mg g^−1^.^131^ A Ni-MOF composite with graphene oxide (Ni-MOF/GO) showed an adsorption capacity of 2489 mg g^−1^ for Congo Red (CR) dye, far greater than previous studies. This composite had a mesoporous structure (2 to 50 nm) with 69.6 m^2^ g^−1^ BET surface area.^132^ The degradation of heavy metals with MOF catalysts is depicted in Fig. 16.

### 4.2 MOFs as photocatalysts for the removal of dyes and pigments

For various cationic colorants such as Methylene Blue (MB), MOF-545/PCN-222 (Zr-metalloporphyrin MOF) has shown effective adsorption rates. The size of the particle and the surface area of the prepared MOF are 3.2 nm and 2336 m^2^ g^−1^, respectively. The adsorption capacity shown by PCN-222 for MB is high, *i.e.* 906 mg g^−1^.^134^ A new composite UiO-66-(OH)_2_ with graphene oxide was investigated for the removal of methylene blue dye. The surface area and volume of UiO-66-(OH)_2_/GO composite were 239.50 m^2^ g^−1^ and 0.0464 cm^3^ g^−1^ respectively. It showed that the MB removal efficacy is up to 99.96%; hence, it is considered as an efficient MB adsorbent.^135^ MIL-101(Fe), an iron-based MOF, was used for Methylene blue removal from wastewater. MIL-101(Fe) with a pore size of 1.66 nm showed 58.8 mg g^−1^ adsorption capacity for MB at pH 9.^136^ A novel zinc MOF (ABim-Zn-MOF) was prepared by using 1-benzylimidazole as a ligand and hexanedioic acid as a linker and investigated for the adsorption of MB dye from wastewater. The particle size, particle volume and specific surface of this MOF calculated using BET analysis were 13.43 nm, 0.68 cm^2^ g^−1^ and 1.40 m^2^ g^−1^. It shows 174.64 mg g^−1^ adsorption capacity for MB.^137^ Cu-BTC-1 MOF synthesized *via* solvothermal technique was considered to be a very efficient MOF for the methylene blue dye removal. At the optimum temperature (298 K), it nearly adsorbs 98.1% of MB dye. On equilibrium establishment, the removal efficacy was increased to 98.6%.^138^. [Ni(HBTC)(Bimb)]_*n*_, a two-dimensional Ni-MOF, displays good removal efficiency for RhB (cationic dye), *i.e.* 87% and 227 mg g^−1^ adsorption capacity.^139^ A novel composite of MOF-5 with graphene oxide (MOF-5@GO) has shown high removal rates for Rhodamine B from wastewater. Within 5 minutes of contact time, the percentage removal was 98.8%, and after 10 minutes, the rate is 99.6%. Therefore, these MOF composites are far superior to pristine MOF-5.^140^ The MIL-53(Al) adsorption performance was outstanding. The specific surface area and particle volume calculated through BET analysis were 610.5 m^2^ g^−1^ and 0.97 cm^3^ g^−1^, respectively. At a temperature of 29.8 °C, the uptake capacity for RhB reaches up to 1547 mg g^−1^, and within a contact time of 120 minutes, the removal efficacy surpassed 90%.^141^ Iron MOFs were considered to be very effective and economic in eliminating Rhodamine B (RhB) from waste water. The Specific surface area of the mesoporous MOF (8.6 nm particle diameter) calculated using BET analysis was 21.48 m^2^ g^−1^. Within a contact time of 4 h. and at pH 6, the adsorption ability of the prepared Fe-MOF for RhB was 135 mg g^−1^.^142^ BUT-8(Cr), a chromium-based MOF, showed excellent adsorption uptake for Rhodamine B, *i.e.* 811.7 mg g^−1^, far greater than already existing MOF materials. Even after 7 consecutive recycles, the removal percentage was still high, *i.e.* 94.5%.^143^

**Fig. 17:**
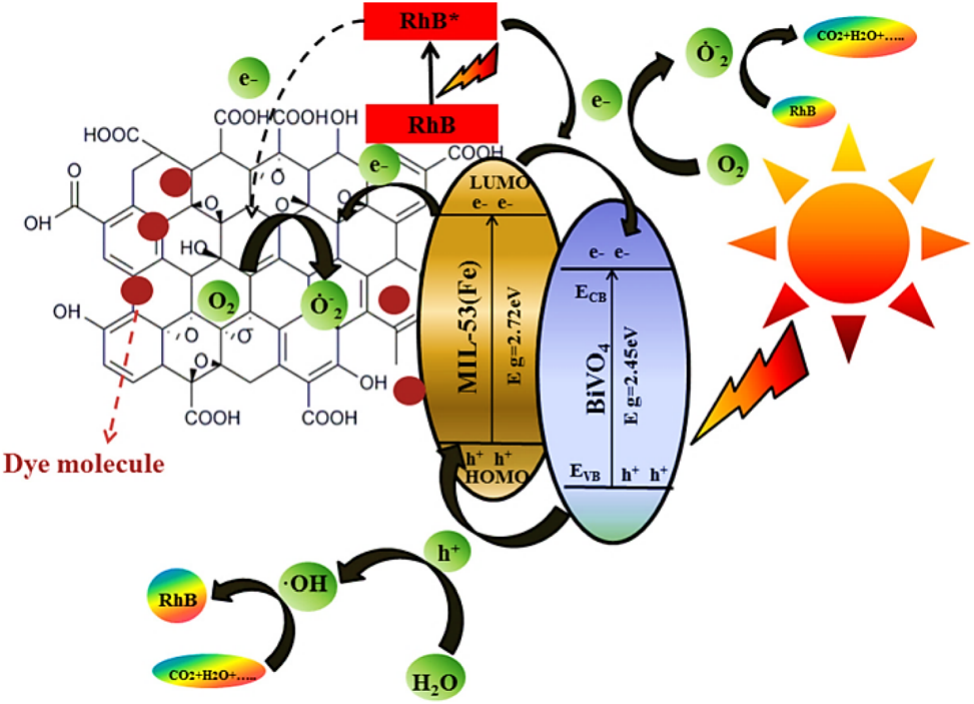
Degradation of Rhodamine B (RhB) dye using BiVO_4_/MOF/GO nanocomposites. Reproduced with permission from ref. 159. Copyright (2020) Elsevier.

One-step precipitation method was used to prepare flaky boat-shaped crystals of Co-MOF with 500 nm pore size, showing high adsorption capacity, *i.e.* 1019 mg g^−1^ for Congo Red dye. The robust p–p stacking and electrostatic interactions were responsible for this high uptake of Congo red by Co-MOFs.^144^ Aluminum fumarate MOF (AlF-MOF) and its composite AlF-MOF/GO and AlF-MOF/rGO were synthesized and investigated for their adsorption behavior towards Congo red dye. The *S*_BET_ (BET surface areas) of prepared MOF and its composites were 973.39, 917.79 and 951.88 m^2^ g^−1^, respectively. The composites (AlF-MOF/GO and AlF-MOF/rGO) adsorb the CR dye more effectively than AlF-MOF showing the adsorption capacities of 102.04 and 178.57 mg g^−1^, while that of AlF-MOF is only 93.45 mg g^−1^.^145^ La-MOF-NH_2_@Fe_3_O_4_ as an effective, quick, and highly selective adsorbent was applied for the adsorptive removal of Congo red dye. The particle diameter is 16.32 nm (mesoporous structure), providing ample space for dye incorporation within the structure. Only after a contact time of 2 min, the percentage removal is 92.01% and adsorption capacity is 716 mg g^−1^.^146^ A highly porous, bifunctional, 3D Zn-MOF having 102.36 m^2^ g^−1^ BET surface area and 2.94 nm particle size is highly stable and shows an adsorption capacity of 355.16 mg g^−1^ for the CR dye. The CR dye adsorption is primarily because of the sedimentation of big molecules formed due to the hydrogen bonding between μ_3_–OH– and –HNH groups in Zn-MOF and CR dye molecules, respectively.^147^ A stable Cobalt MOF named [Co(api)(nita)] DMF was investigated for the removal of hazardous Reactive Black 5 from wastewater. The percentage removal of Co-MOF was 78.24% with an adsorption uptake of 18.80 mg g^−1^.^148^ TMU-8, a Cd-based MOF with the formula ([Cd_2_(oba)_2_(4-bpdb)_2_]_*n*_·3.5(DMF)), shows an adsorption capacity of 79.36 mg g^−1^ for Reactive Black 5.^149^ The nano porous MIL/CNT (MIL-125(Ti)/carbon nanotube) composites exhibited enhanced photocatalytic degradation for RB5 because of the synergistic action of carbon nanotubes. The RB5 elimination rate shown by MIL-125(Ti) was 0.0015 mg L^−1^ min^−1^ and for MIL-CNT (0.01) and MIL-CNT (0.03), the values were 0.0019 and 0.0024 mg L^−1^ min^−1^, respectively.^150^ MIL-101-Cr MOF was investigated for its adsorptive removal of toxic Reactive Black 5 dye. It has a microporous structure with 828 nm pore size and 2410 m^2^ g^−1^ BET surface area. The adsorption uptake of MIL-101-Cr was between 377 and 397 mg g^−1^.^151^ The silver nano-composite Ag@MOF-801/MIL-88A(Fe) was synthesized by templating in MOFs having a particle volume and surface area (*S*_BET_) of 0.16 cm^3^ g^−1^ and 145.2 m^2^ g^−1^. After 30 minutes of exposure to visible light, the resulting Ag@MOF-801/MIL-88A(Fe) nanocomposite showed 91% Reactive Black 5 photocatalytic degradation.^152^ The Cu-MOF/Fe_3_O_4_ composite was found to be an effective adsorbent for the elimination of malachite green^153^ from contaminated water. The MG adsorption rate was higher, *i.e.* 113.67 mg g^−1^, compared to already reported adsorbents.^154^ Organic–inorganic Zn-MOFs were considered to be the most efficient adsorbents for toxic Malachite green removal, as it had shown the adsorption capacity of 953.14 mg g^−1^. The surface area and particle size of CZM nanoparticles were 1820.7 m^2^ g^−1^ and 1.73 nm respectively. After adsorption, there is a decrease in both surface area and particle size, *i.e.* 953 m^2^ g^−1^ and 1.70 nm indicating the proper filling of the MG dye in microporous voids of CZM.^155^ The Fe-BTC MOF was employed as an adsorbent to remove the malachite green dye from wastewater. The BET surface area of the prepared MOF was 443 m^2^ g^−1^. The isotherm model conformed to the Langmuir isotherm, exhibiting an MG uptake of 177 mg g^−1^.^156^ ZIF-8@ZnAl-LDH, a high-porosity MOF composite synthesized by the *in situ* development of ZIF-8 (zeolite imidazole framework) on ZnAl-LDH (Zn layered double hydroxide), showed a malachite green uptake up to 194.5 mg g^−1^. Within an exposure time of 3 h, the MG removal rate was 98%. The synthesized MOF composite has a high specific surface area (963 m^2^ g^−1^) compared to simple ZnAl-LDH whose surface area was only 210 m^2^ g^−1^.^157^ Another zeolitic imidazolate framework, ZIF-67, showed an adsorption capacity of 2430 mg g^−1^ for Malachite green just at 293 K temperature, and it can be increased further by increasing the temperature.^158^ Chen *et al.* (2020) developed a new BiVO_4_/MOF/GO ternary photocatalyst by hydrothermal methods. The as-prepared BiVO_4_/MOF/GO composites showed outstanding photocatalytic activity for the degradation of Rhodamine B (RhB) under visible light irradiation, as graphically represented in Fig. 17.^159^

### 4.3 MOFs as photocatalysts for the removal of organic compounds

**Fig. 18:**
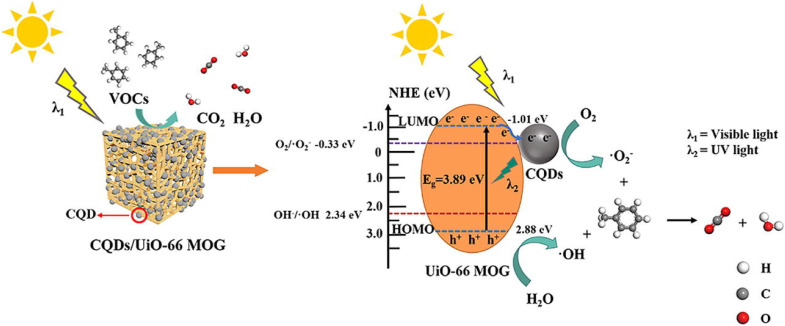
Photocatalytic degradation of toluene by CQDs/UiO-66 MOG composites. Reprinted with permission from ref. 170. Copyright (2022) Elsevier.

A highly stable HKUST-1 MOF was selected as an adsorbent for paranitrophenol (PNP) removal due to its adsorption capability of 400 mg g^−1^. This high adsorption was considered due to the strong interactions between the nitro (–NO_2_) group of PNP and the metal sites in HKUST-1 along with interactions of PNP Benzene rings with HKUST-1.^160^ UiO-66-NH_2_ was used for effective removal of a number of phenol derivatives such as 2,4,6-trinitrotoluene (TNT), 2,4-dinitrotoluene (2,4-DNT), picric acid (PA), trinitroresorcinol (TNR) and 2,4-dinitrophenol (2,4-DNP) showing 0.0005, 0.002, 0.0225, 0.024, and 0.0296 g g^−1^ adsorption capacities, respectively. The strong hydrogen bonding between these phenol derivatives and UiO-66-NH_2_ was considered to be the main cause for such high adsorption of pollutants.^161^ MOF-199 (Cu-based MOF) and ZIF-8 (Zr-based MOF) were synthesized and their adsorption behaviors towards the removal of phenol and paranitrophenol (PNP) were examined. The results showed that MOF-199 has higher removal rates for both phenol and PNP, *i.e.* 79.55% and 89.3% while ZIF-8 has removal rates of 65.5% for phenol and 77% for PNP.^162^ An Al-MOF/SA-CS (aluminum MOF/sodium alginate-chitosan) composite with a larger BET surface area of 687.54 m^2^ g^−1^ was employed for the adsorptive removal of bisphenol A from water. With the increase in the BPA concentration up to 120 ppm, the adsorption capacity of the composite increases to 136.9 mg g^−1^.^163^ NH_2_-MIL-88B having 414 m^2^ g^−1^ BET surface area and hexagonal structure was considered to be a competent adsorbent for 2,4,6-trinitrophenol (TNP) in a liquid medium as it showed an adsorption capability of 163.66 mg g^−1^. The adsorption mechanism of TNP by NH_2_-MIL-88B is probably attributed to hydrogen bonding, as well as complex formation between the hydroxyl groups in TNP and the unsaturated Fe(iii) sites present on NH_2_-MIL-88B surfaces.^164^ Zn-based MOF synthesized using a terephthalic acid ligand was investigated for their adsorption behavior towards anthracene and naphthalene. Zn-BDC MOF showed a higher removal rate of 97% for naphthalene than for anthracene, which showed only 50% removal rate. The remarkable percentage removal observed with naphthalene is because of more secure fitting within the holes of MOF particles, given that molecules of naphthalene experience less steric hindrance than anthracene.^165^ Highly porous, nanosized Zr-based MOFs UiO-66 and NH_2_-UiO-66 with 1420 and 985 m^2^ g^−1^ BET surface area and 7.56 nm and 3.56 nm particle size appeared to be highly effective in eliminating chrysene and anthracene from aqueous solutions. Within a contact time of 25–30 minutes, UiO-66 and NH_2_-UiO-66 achieved removal rates of 97.9% and 95.7% for chrysene, and 98.6% and 96.4% for anthracene, respectively.^166^ Moreover, NH_2_-UiO-66 has shown higher removal rates of 87.2 and 89.1% for phenanthrene and naphthalene, and can be reused for at least seven times.^167^ The zeolitic imidazole framework composite with Fe_3_O_4_ nanoparticles (Fe_3_O_4_@ZIF-8) having a surface area of 942 m^2^ g^−1^ and a particle diameter of 3.17 nm was investigated for toluene and benzene adsorptive removal. The MOF composite adsorption increases with the increase in temperature and at a temperature of 50 °C, the maximum adsorption capacities for toluene and benzene were 133 and 148 mg g^−1^ along with 93% and 98% removal efficiencies, respectively.^168^ The microwave-assisted solvothermal method was employed for the synthesis of two highly porous iron MOFs, MIL-88(Fe) and NH_2_-MIL-88(Fe), having BET surface areas of 1240 and 941 m^2^ g^−1^, respectively. Within a contact time of 40 min, MIL-88(Fe) and NH_2_-MIL-88(Fe) have shown removal rates of 99% and 96% for pyrene.^169^ Yu *et al.* (2022) used a quasi *in situ* approach to develop a series of carbon quantum dots (CQDs) adorned UiO-66 metal–organic-framework gel (MOG) composites with hierarchical pore architectures. CQDs/UiO-66 MOG was then employed to photocatalyze the breakdown of toluene under sunshine. Using 0.5 wt% CQDs/UiO-66 MOG as the photocatalyst resulted in a rather high CO_2_ conversion ratio (85%) for toluene as shown in Fig. 18.^170^

### 4.4 MOFs as photocatalysts for the removal of pesticides and herbicides

**Fig. 19:**
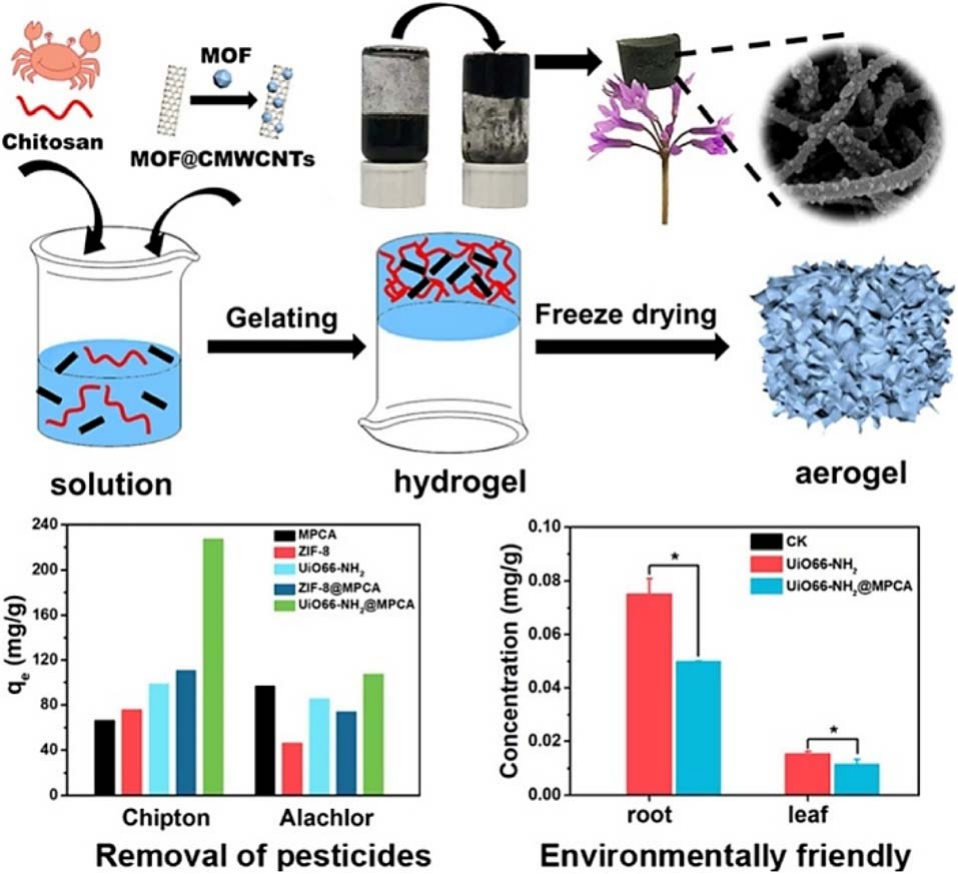
Photocatalytic degradation of pesticides using MOF@MPCA. Reproduced with permission from ref. 175. Copyright (2021) Elsevier.

The octahedral, highly crystalline and mesoporous MIL-101(Cr) with 2600 m^2^ g^−1^ specific surface area was employed for diazinon removal from contaminated water using a fixed-bed method. MIL-101(Cr) showed the highest removal rate of 92.5% at a neutral pH.^171^ Two novel Zn- and Co-based zeolitic imidazolate frameworks, namely ZIF-8 and ZIF-67, were synthesized in order to investigate their adsorption behavior for two hazardous organophosphorus pesticides such as ethion and prothiofos. The results revealed that ZIF-8 showed higher adsorption capacities for both pesticides, *i.e.* 366.7 and 279.3 mg g^−1^ for prothiofos and ethion, while ZIF-67 shows relatively less adsorption capacities, *i.e.* 261.1 and 210.8 mg g^−1^, respectively. This indicated that both pesticides have greater affinities with zinc ions rather than cobalt ions.^172^ A 3D CaFu MOF with 2308.03 m^2^ g^−1^ BET surface area was used for the removal of highly consumable imidacloprid pesticide through adsorption. The particle size of CaFu MOF was 30 nm with truncated octahedron shape and good uniformity. The adsorption capacity achieved using this MOF was 467.2 mg g^−1^ and the percentage removal was 98.3% from 1 to 5 h.^114^ A Sn-MOF with a high surface area of 897.6 m^2^ g^−1^ was used for the removal of Diazinon pesticide from aqueous media. The maximum adsorption capacity of the Sn-MOF was 587.3 mg g^−1^, and it was achieved when the solution pH was slightly acidic, *i.e.* 6.^173^ An innovative efficient adsorbent, Fe_3_O_4_@SiO_2_@UiO-67, was used for the concurrent elimination of glyphosate pesticide. Zr–OH groups of the synthesized adsorbent are strongly attracted to the phosphate group, resulting in higher glyphosate adsorption rates. It boasts the adsorption capacity to 256.5 mg g^−1^, with a minimum detection limit of 0.093 mg L^−1^, and can be reused multiple times. This suggests that the adsorbent has amalgamated the benefits inherent in its individual components.^174^ Liang *et al.* (2021) developed a novel method of loading two MOFs (ZIF-8 or UiO-66-NH_2_) on carbon nanotube aerogels (MPCA) by *in situ* nucleation and growth to reduce the secondary risks of using MOF nanoparticles as adsorbents. The synthesized material was used to remove pesticides from water. MOF@MPCA displayed high hydrophilia, compression resilience, and thermostability. Comparing the adsorption capacity of MOF@MPCA with single MOF nanoparticles demonstrated that MOF@MPCA has higher adsorption efficiencies of chipton and alachlor, proving the mutual effect of MOFs and MPCA, as depicted in Fig. 19.^175^

### 4.5 MOFs as photocatalysts for the removal of gases and vapours

A 3D In(iii)-based MOF (In-ABDC) with a diamond-like network and containing a pseudo-tetrahedral node [In(O_2_CR)_4_]^−^ was synthesized and investigated for CO_2_ adsorption. The surface area of the prepared MOF calculated using BET was 307 m^2^ g^−1^ with 0.38 cm^3^ g^−1^ pore volume. The CO_2_ adsorption rate varies greatly with the temperature, *i.e.* 81.3, 31.1 and 16.4 cm^3^ g^−1^ at temperatures of 196, 273 and 298 K, respectively.^176^ A new Mg/Zn-MOF-74 (bimetallic) was synthesized through one-pot synthesis using Zn and Mg metals in a ratio of 75/25. Under 1 bar pressure and 273 K temperature, the synthesized MOF has shown an excellent adsorption rate for CO_2_, *i.e.* 128 cm^3^ g^−1^ and it can be reused for five consecutive cycles.^177^ Two Cd and Cu-based MOFs with oxalamide functionality have been synthesized solvothermally. Among them, the Cu-OATA-MOF has shown higher adsorption for CO_2_, *i.e.* 138 cm^3^ g^−1^ and 50.09 cm^3^ g^−1^ at 273 K and 298 K, respectively. While for Cd-OATA-MOF, the adsorption capacities were 60.57 and 11.40 cm^3^ g^−1^ at 273 and 298 K, respectively, much less than that of Cu-oxalamide MOF.^178^ Ni-MOF-74 has been investigated for its behavior towards CO_2_ adsorption. The adsorption capacities shown by this MOF were 8.29 and 6.61 mmol g^−1^ at temperatures of 273 and 298 K and a pressure of 1 bar. The impressive capacity for CO_2_ capture is attributed to the plentiful adsorption sites, primarily originating from Ni^2+^ ions, and the small micropore channels, primarily resulting from the cage structure formed by the coordination of Ni^2+^ ions with organic ligands.^179^ Cu_1.5_Mg_1.5_(btc)_2_, a bimetallic MOF, was investigated for its CO_2_ adsorption and the results were compared with parent MOFs, *i.e.* Cu-btc and Mg-btc MOFs. The CO_2_ adsorption capacity exhibited by bimetallic MOFs shows a significant improvement, reaching 23.85 mmol g^−1^ in comparison to Cu and Mg-btc MOFs, showing 5.95 and 4.57 mmol g^−1^ adsorption capacities. This underscores the pivotal role of the central metals within the MOF structure in facilitating CO_2_ adsorption.^180^ A Cu-MOF with inorganic anion pillars (SIFSIX and SiF_6_^2−^) and SIFSIX-2-Cu-i was employed for the adsorptive removal of sulfur dioxide from the atmosphere. At 1 bar (atmospheric pressure), the SO_2_ adsorption achieved was 11.01 mmol g^−1^, while at low pressures, *i.e.* 0.002 and 0.01 bar, the adsorption capacity decreased to 2.31 and 4.16 mmol g^−1^, respectively.^181^ The SO_2_ adsorption has been studied using M-MOF-74, where M belongs to Mg, Zn, Co, and Ni metals. The surface areas calculated using BET analysis for Mg-MOF-74, Co-MOF-74, Ni-MOF-74 and Zn-MOF-74 were 1078, 1077, 913 and 774 m^2^ g^−1^, respectively. The results indicated that among all the MOFs, Mg-MOF-74 was adsorbed more effectively with a bonding energy greater than 90 kJ mol^−1^, and Zn-MOF-74 showed a binding energy of 70 kJ mol^−1^.^182^ The adsorption behavior of highly porous PAF-302 COF towards SO_2_ uptake was investigated by employing GCMC simulations. The BET surface area of the prepared COF was 5600 m^2^ g^−1^ with 12.4 Å particle size. The maximum SO_2_ uptake shown by PAF-302 was 50.69 mmol g^−1^, which is much greater than that of earlier reported adsorbents (activated carbons and MILs).^183^ At ambient temperature, ZnO/Zn-MOF nanocomposites were employed to convert hazardous SO_2_ gas into sulfates with a surface area ranging from 12.4 to 20.6 m^2^ g^−1^. Under standard conditions, the SO_2_ uptake capacity with these nano composites was 31.0 mg g^−1^. The adsorption capacity decreased as the temperature increased because adsorbed water and molecular oxygen were removed, which were necessary for SO_2_ oxidation to occur following the adsorption process.^184^ SO_2_ present in flue gas and other gases was effectively removed using ELM-12, a microporous MOF. Activated ELM-12 was found to have a specific surface area of 706 m^2^ g^−1^ and a pore volume of 0.26 cm^3^ g^−1^. At 1 bar atmospheric pressure and 25 °C temperature, the SO_2_ uptake from a 10 : 90 mixture by ELM-12 was 61.2 cm^3^ g^−1^.^185^ UiO-66 modified using HAc resulted in enhanced specific surface area, *i.e.* 1450.1 cm^2^ g^−1^. UiO-66-1.0HAc exhibited the maximum adsorption capability for benzene at 25 °C (367.13 mg g^−1^). UiO-66-2.0HAc demonstrated a remarkable 93% increase in toluene adsorption capacity, reaching 410.21 mg g^−1^ at the same temperature, surpassing the original counterpart.^186^ The synthesis of bio-MOF-11, an adenine-based MOF with a surface area of 580 m^2^ g^−1^, was carried out. Subsequently, its adsorption capabilities for four common VOCs (methyl alcohol, dimethylketone, benzene, and toluene) were systematically investigated. It showed varying adsorption capacities ranging from 0.73 to 3.57 mmol g^−1^. The order of adsorption efficiency was observed as follows: toluene < benzene < dimethylketone < methyl alcohol.^187^ The adsorption behaviors of MIL-101 (Cr-based MOF) and its composite with graphite oxide (MIL-101@GO) having high BET SSA, *i.e.* 2920 m^2^ g^−1^ and 3439 m^2^ g^−1^, were investigated towards benzene, ethyl benzene and toluene. MIL-101@GO appeared to be more efficient towards adsorption of the above-mentioned aromatics, 1.8 to 6.0 times higher than that of commonly used adsorbents. Moreover, the composite demonstrates a higher adsorption rate for benzene, *i.e.* 20 mmol g^−1^, and then decrease in the order of toluene (16.6 mmol g^−1^) > ethylbenzene (13.6 mmol g^−1^).^188^

**Fig. 20:**
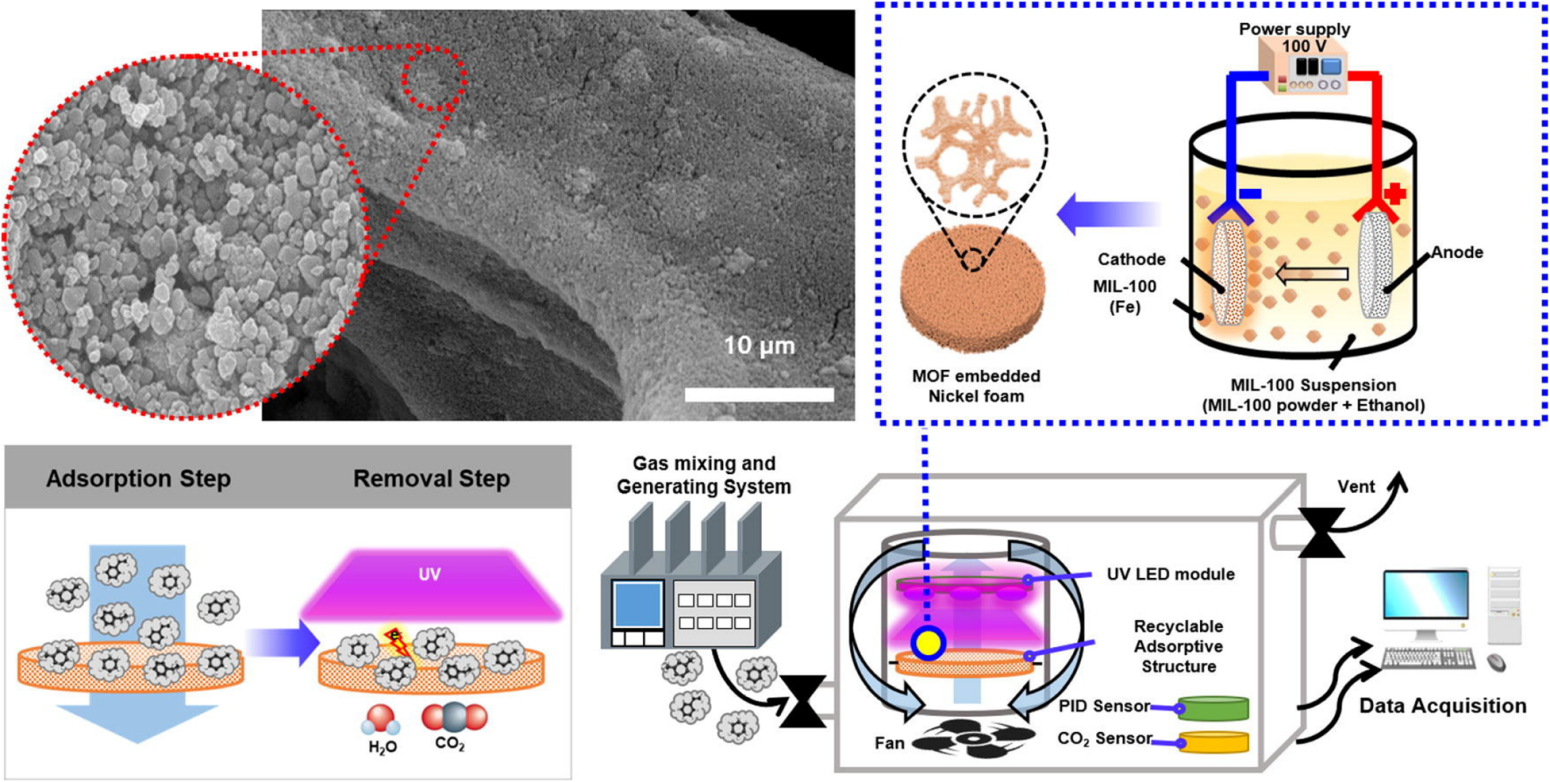
Adsorption and photodegradation of volatile organic compounds (VOCs) using MIL-100(Fe) under UV light. Reproduced with permission from ref. 194. Copyright (2022) Elsevier.

The Fe-based MOFs MIL-53, MIL-88B, and MIL-101 were chosen as the photocatalysts for the oxidation of NO_*x*_. Overall, MIL-101 demonstrated excellent photocatalytic performance, showing 76% and 61% efficiency in NO conversion in both sunlight and UV-filtered illumination, which is because of its remarkable surface area, significant pore size volume, and coordinately unsaturated Fe spots.^189^ The MOF UiO-66-NH_2_ was utilized for the elimination of NO_2_ from the air. The existence of the amine group in this MOF significantly enhances the removal rate, leading to remarkable removal capacities exceeding 1.4 g of nitrogen dioxide per gram of MOF.^190^ A crystalline Ni-MOF catalyst having SBET in the range of 27.8–32.2 m^2^ g^−1^ was used for the Selective Catalytic Reduction of NO_*x*_ with NH_3_ (SCR-NH_3_). Upon activation at 220 °C, this Ni-MOF catalyst demonstrated a NO conversion efficiency exceeding 92% across the temperature spectrum of 275–440 °C.^191^ The Cu-BTC MOF, recognized as a remarkably efficient MOF, was manufactured and integrated with NTP (non-thermal plasma), resulting in an outstanding efficacy of 97.86% for nitrogen oxide removal. Cu-BTC-NTP exhibits a cubic octahedron morphology with a diameter in the range of 10–30 μm.^192^ NO_2_ was reactively adsorbed onto the surface of vanadium MOF, MFM-330(V). During NO_2_ adsorption by MFM-300(V), V(iii) was oxidized to V(iv) simultaneously by reducing the adsorbed nitrogen dioxide to nitrogen oxide and by releasing water through deprotonating the –OH group of MFM-330(V). The effective arrangement of {N_2_O_4_·NO_2_}_∞_ chains within the MFM-300(V) pores leads to notable NO_2_ adsorption (13.0 mmol g^−1^) at 25 °C and 1.0 bar, and it can be reused multiple times.^193^ The research by Lee *et al.* (2022) used a photocatalytic metal organic framework (MOF) filter (PMF) based on MIL-100(Fe) with a particle size of ∼160 nm to filter indoor VOCs by a hydrothermal method. The electrophoretic deposition of MOF nanoparticles on a porous nickel foam produced PMF. It retained an even distribution of MOF nanoparticles without the need of uncoating on a nickel foam with a diameter of 10 cm. Because of the special features of the large specific surface area and photocatalytic function of MIL-100(Fe), the PMF was effective in VOC adsorption and photodegradation upon UV light exposure as described in Fig. 20.^194^

### 4.6 MOFs as photocatalysts for the removal of waterborne pathogens

**Fig. 21:**
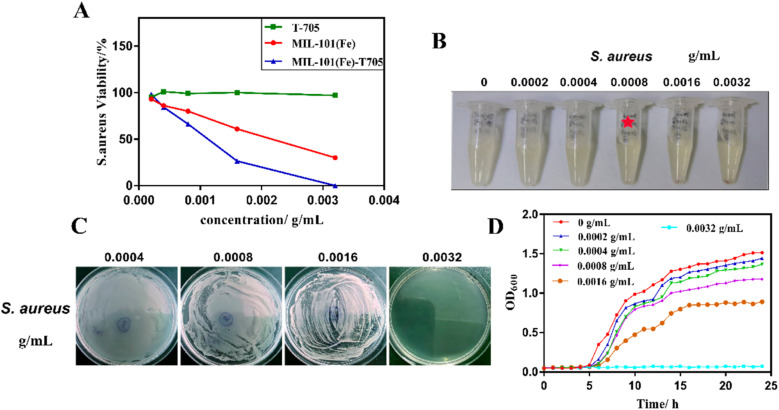
*In vitro* antibacterial study of MIL-101(Fe)-T705. Reprinted with permission from ref. 202. Copyright (2022) MDPI.

An amino-modified Cu-based MOF (NH_2_-Cu-MOF) was prepared and its effectiveness in removing endotoxin (contaminant excreted by cyanobacteria) from water bodies was explored. The adsorbent exhibited a degradation rate of over 90% for endotoxin, with a maximum adsorption capacity exceeding 2500 Eu mg^−1^.^195^ A metal–organic framework incorporating zinc and featuring hydrazinebenzoate linkers was assessed for its antimicrobial properties against the Gram +ve bacterium *S. aureus*. The MOF, when distributed in the culture medium, has a half-maximum effective dose of roughly 20 mg L^−1^ and suppresses both metabolic activity and bacterial proliferation.^196^ Zn^2+^ salt and azelaic acid, both of which have fascinating antibacterial properties, were used to produce BioMIL-5 (bioactive and biocompatible MOF). Because of the rapid disintegration and component excretion rate of Bio-MOF (56.7 ± 3.9% of Zn^2+^ being released within 10 weeks), the Zn^2+^ ions released throughout the process demonstrated strong antibacterial capabilities against *S. epidermidis* and *S. aureus*.^197^ The Zn-PDA MOF, prepared using Zn(NO_3_)_2_·6H_2_O and ligand 2,6-PDA (pyridine dicarboxylic acid), revealed strong antimicrobial activity against *S. aureus*, *Acinetobacter baumannii*, *Bacillus subtilis*, *Klebsiella pneumonia*, *Salmonella enterica*, and *E. coli*. It demonstrated average inhibition diameters ranging from 8.6 to 17 mm and a Minimum Inhibitory Concentration (MIC) value between 300 and 308 μg mL^−1^ due to the small particle size and elevated surface area of the MOF.^198^ BioMOF-1, [Ag_4_(μ_3_-PTA)_2_(μ-PTA)_2_(μ_4_-pma) (H_2_O)_2_]*n*·6*n*H_2_O, was employed in order to investigate its antiviral efficacy against HAdV-36 (human adenovirus 36). When tested at a concentration of 50 μM for half an hour, it exhibited reduction factors of ≥4.00 log10, indicating a significant decrease in virus infection, suggesting a potential inhibitory effect of BioMOF-1 on adenovirus.^199^ Thio@MIL-125-NH_2_@CMC, a newly developed eco-friendly active composite, formed through post-synthetic modification, involves MIL-125-NH_2_ (Ti-MOF) and carboxymethyl cellulose improved with thiophene. The synthesized composite exhibited significant antiviral efficacy against HSV1 (68.89%) and COX B4 (39.60%), which appeared as promising antiviral agents against both viruses.^200^ Three Metal–Organic Frameworks based on zinc, namely MOF-5, IRMOF-3 and Zn-BTC, were prepared to investigate their antibacterial effects on *S. aureus*, *L. monocytogenes*, *E. coli*, and *S. lentus*. The observed MIC values for these bacteria ranged from 100 to 250 μg mL^−1^.^201^ The MIL-101(Fe)-T705 complex, prepared using MIL-101(Fe) MOF and favipiravir (T-705) drug, having 116.7 m^2^ g^−1^ BET surface area and 1.52 nm particle size was employed in order to study its antiviral properties. MIL-101(Fe)-T705, displaying superior biosafety over 12–72 hours, exhibited potent antiviral effects at concentrations (0.1 to 3 μg mL^−1^), surpassing MIL-101(Fe) and T-705 in inhibiting influenza, as shown in Fig. 21.^202^

### 4.7 MOFs as photocatalysts for the removal of radioactive elements

**Fig. 22:**
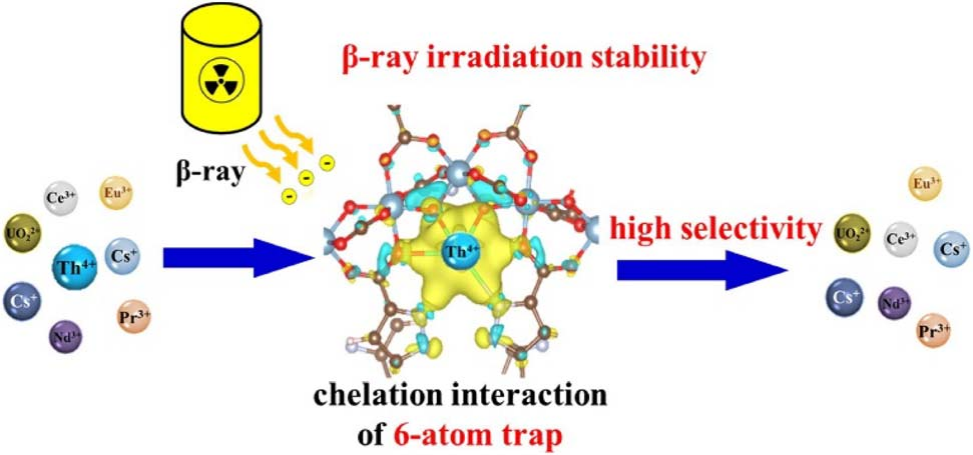
MOF-303 as an effective β-ray irradiation-resistant trap for capturing Th(iv) ions. Reprinted with permission from ref. 210. Copyright (2022) Elsevier.

The potential of ZIF-8@Fe and ZIF-67@Fe as promising adsorbents for the extraction and separation of uranium-VI ions from water bodies was investigated. These materials demonstrated impressive adsorption capabilities of 277.8 mg g^−1^ and 292 mg g^−1^ respectively, along with high selectivity for U-VI ions at a pH of 4.5. The synergistic interaction between the metallic Fe(ii) component and N-donor coordination sites was identified as the key factor contributing to the maximum uptake of U(vi) by ZIF-8@Fe and ZIF-67@Fe.^203,204^ Co-SLUG-35 MOF, cationic in nature, has been used for the extraction of U-VI from seawater and basic solutions. The U-VI ions (*via* anionic exchange) completely replace EDS^2−^ anions from the alkaline solution of U-VI, showing adsorption capacities of 118 mg g^−1^ and 1.05 mg g^−1^ with U-VI ions from one liter of 5.35 ppb seawater.^205^ The properties of Zn-MOF-74 were enhanced using coumarin functionality by embedding it on the unsaturated Zn(ii) coordination sites, resulting in a collection of Zn-MOF-74 materials modified with coumarin. Zn-MOF-74 having both mesoporous and microporous sizes, showing remarkable U-VI ion uptake with an adsorption capacity of 360 mg g^−1^ at an optimum pH 4.^206^ For the effective adsorption approach of removing U(vi) from liquid phases, a MOF structure with Lewis basic groups and UTSA-76 encoding was created. The Langmuir model provided the best fit for the isothermal adsorption, and its maximum adsorption capacity was determined to be 91.31 mg g^−1^. In a solution with different concentrations (0.25 ppm–12 ppm), a mere 5 mg dose of UTSA-7 was used, and after 45 minutes of contact, the UO_2_^2+^ ion removal rate was 98%, demonstrating the adsorbent's great efficiency in removing uranium.^207^ For selective adsorption and capture of Thorium ions from water, MOF MIL-100(Al) with a particle size of 3 to 4 nm was analyzed. The MIL-100(Al) MOF demonstrates a high removal competence of around 95% for Th^4+^ ions. The preference of MIL-100(Al) for Th^4+^ ions was credited to the chemical reactivity of Th^4+^ ions rather than the Al-MOF structure.^208^ For the adsorption of Th(iv) from less acidic solutions, the adsorption behavior of UiO-66 along with its analogues UiO-66-COOH and UiO-66-(COOH)_2_ was examined. UiO-66 has a higher BET surface area of 1000 m^2^ g^−1^ than that of its derivatives, which have SBET of 540 m^2^ g^−1^ and 250 m^2^ g^−1^, respectively. With an adsorption capability of 360 mg g^−1^ for Th^6+^, UiO-66-(COOH)_2_ surpasses UiO-66-COOH's capacity by two times and significantly higher than that of UiO-66.^209^ Highly stable MOF-303 (an Al-based MOF) containing massive ion traps was employed for selective capturing of thorium ions (Th^4+^) from water. The Th^4+^ uptake was extremely high, *i.e.* 461.7 mg g^−1^ and this was attributed to the distinctive chelating interactions by the ion traps. MOF-303 also exhibits remarkable separation coefficients, *i.e.* 97.6 for Th^4+^/Pr^3+^, 97.3 for Th^4+^/Eu^3+^and 81.3 for Th^4+^/Nd^3+^, as depicted in Fig. 22.^210^

**Fig. 23:**
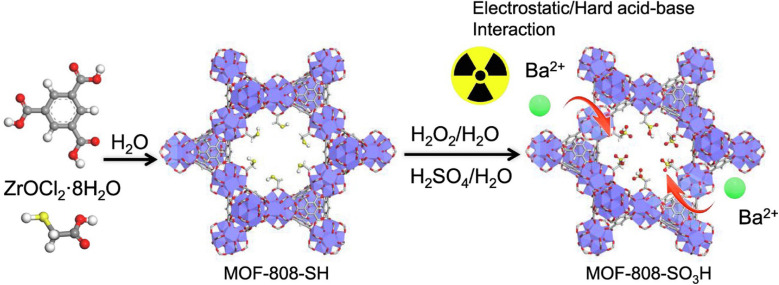
MOF-808-SO_3_H as a photocatalyst for the removal of radioactive elements. Reproduced with permission from ref. 216. Copyright (2022) Elsevier.

A stable aluminium-based metal organic framework, CAU-1 NH_2_, with 550 m^2^ g^−1^ BET surface area was investigated for thorium removal from contaminated water. The MOF represented a high thorium uptake capacity of approximately 404 mg g^−1^, and due to its stability, it can be reused many times.^211^ Another 3D-Al-based MOF is named Zn (L_1_)_0.5_(DMF)_2_ (AZOXDC), where H_2_AZOXDC stands for 4,4′-azoxydibenzoic acid and L_1_ stands for *N*,*N*′-dipyridin-4-ylterephthalamide. The synthesized MOF having 7.76 m^2^ g^−1^ BET surface area has removed 99.8% of Th^4+^ ions from 100 ppm Th(iv)-containing solution within a contact time of just 10 minutes, which appeared to be a highly efficient MOF for thorium removal.^212^ In another study, MOF-808 with a sulfate functionality (MOF-808-SO_4_) and a Cr-based MOF with a sulfonic acid functionality [MIL-101-SO_3_H(Cr)] were observed to show higher barium uptakes of 131.1 mg g^−1^ and 70.5 mg g^−1^, respectively which represent a 328-fold increase and a 60-fold increase compared to the original MOFs.^213^ Two Zr-based MOFs, Zr-MSA-SO_3_H and Zr-DMSA-SO_3_H, were synthesized and investigated for their adsorption behaviour towards radioactive Ba^2+^ ion removal. Because of the nano range particle size and elevated concentration of active SO_3_H groups, Zr-DMSA-SO_3_H shows swift achievement of adsorption equilibrium within 30 minutes and boosts a superior adsorption capacity for Ba^2+^ at 224.0 mg g^−1^, surpassing other MOFs reported in the literature.^214^ The adsorption behaviour of another Zr-based MOF, Zr-BDC-NH_2_-SO_4_, towards Ba^2+^ ion removal was investigated. The surface area of the synthesized MOF calculated through BET analysis was 374 m^2^ g^−1^. It shows a notable adsorption capacity of 181.8 mg g^−1^. Furthermore, even at concentrations ten times higher than those of Ba^2+^, it demonstrates remarkable selectivity in the presence of other metal ions.^215^ The extraction of radioactive Ba^2+^ from nuclear waste holds significant importance in safeguarding the environment. The sulfonic acid and sulfate groups are efficient barium chelating groups, hence attaching these functionalities to different MOF materials result in high Ba^2+^ ion uptake. MOF-808 with a sulfonic acid functionality, and MOF-808-SO_3_H with a surface area of 683.8 m^2^ g^−1^ and a pore volume of 0.472 were employed as adsorbents for the excellent capture of Ba^2+^ ions. The prepared MOF showed a Ba^2+^ ion uptake of 152.0 mg g^−1^. Fig. 23 shows the strong capacity of the MOF for capturing Ba^2+^ ions, which was attributed to the electrostatic and Schiff acid–base interactions.^216^

### 4.8 MOFs as photocatalysts for the removal of inorganic ions

**Fig. 24:**
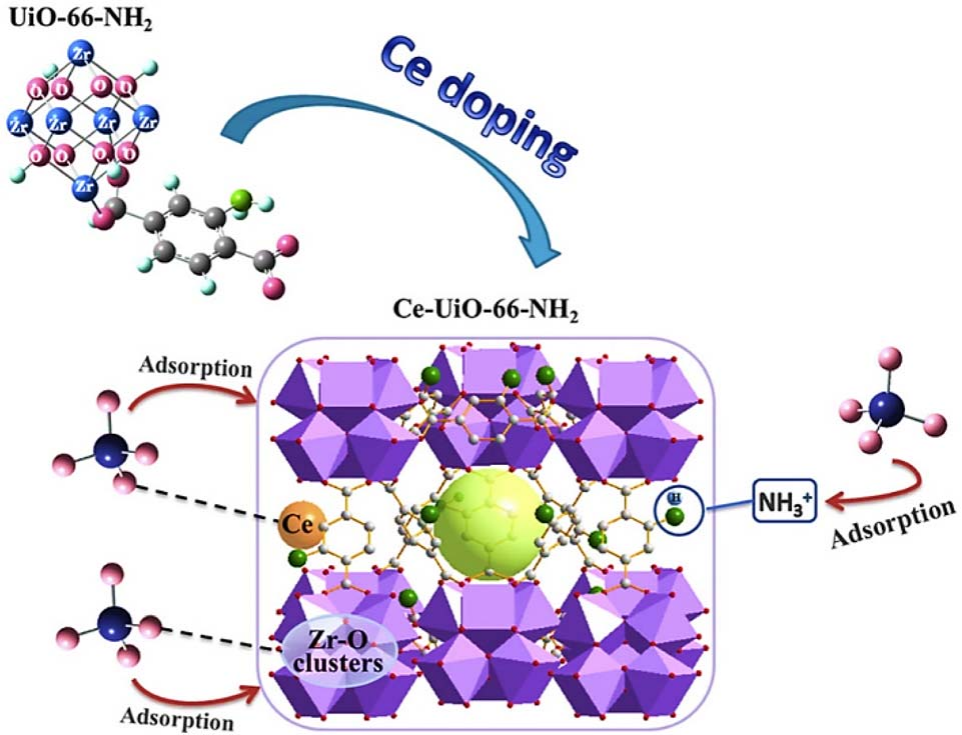
Ce-UiO-66-NH_2_ MOF as a photocatalyst for the removal of phosphate ions from water. Adopted with permission from ref. 222. Copyright (2020) Elsevier.

A Zn-BDC MOF [MOF-5(1)] and its derivative with tri-ethylamine [MOF-5(2)] having BET surface areas of 988.3 m^2^ g^−1^ and 1283.2 m^2^ g^−1^ were synthesized and investigated for nitrate adsorptive removal from wastewater. The particle sizes of MOF-5(1) and MOF-5(2) were 250 nm and 100 nm respectively, according to the SEM results. MOF-5(2) showed a higher nitrate adsorption than that of MOF-5(1), as its surface area decreased by 37.6 m^2^ g^−1^ after adsorbing nitrate ions.^217^ A novel lanthanum-based MOF (LTA-MOF) interconnected with trimeric acid (129 m^2^ g^−1^ BET surface area and 4.063 nm particle width) was synthesized for the adsorption of soluble nitrates (NO_3_^−^) from aqueous solutions. By increasing the adsorbent dose from 0.025 g to 0.15 g, the NO^3−^ adsorption ability increases from 19.72 to 50.09 mg g^−1^.^218^ A 2D-FTA MOF (Fe metal linked with trimeric acid) was employed for the speed removal of soluble fertilizers. The prepared MOF has shown a nitrate adsorption capacity of 55.02 mg g^−1^.^219^ The UiO-66-Sal MOF which is the modified version of UiO-66-NH_2_ was extremely efficient and robust for the uptake of nitrate ions from water. Then 1 gram of the adsorbent was capable enough to remove 95.99% of nitrate ions from 1 liter solution within a contact time of 60 minutes and pH 7. The synergetic effects of the quaternion moiety with the MOF and its high loading on the UiO-66-Sal surface are the key factors for such high removal rate.^220^ Fe-MIL-88B, with 165.45 m^2^ g^−1^ BET surface area and 0.214 cm^3^ g^−1^ pore volume, was used for nitrate removal from water bodies. The synthesized Fe-MOF has shown an adsorption capacity of 92.59 mg g^−1^ for nitrate ions.^221^ A 2D-FTA MOF (Fe metal linked with trimeric acid) was employed for the speedy removal of soluble phosphate fertilizers. The prepared MOF has shown a high phosphate adsorption capacity of 72.34 mg g^−1^.^219^ A novel and remarkably efficient cerium-modified MOF, known as Ce-UiO-66-NH_2_, was employed for the purpose of extracting phosphate from water. Upon incorporating cerium into UiO-66-NH_2_, the resulting Ce-doped MOF demonstrated exceptional efficacy in phosphate adsorption, showcasing a maximum PO_4_^3−^ uptake of 211.86 mg g^−1^ (Fig. 24). The MOF proved its ability to be utilized iteratively for a minimum of five cycles, with the adsorption capacity remaining consistently above 100 mg g^−1^ even after the fifth cycle.^222^

Highly porous MIL-101(Fe) MOF and NH_2_-MIL-101(Fe) were employed for the adsorptive removal of phosphate ions from water and have shown high PO_4_^3−^ removal rates of 92% and 94%, respectively. The surface part of MIL-101(Fe) calculated using BET analysis was 2350 m^2^ g^−1^ and it increased to 2736 m^2^ g^−1^ by the addition of an amine moiety. NH_2_-MIL-101(Fe) has shown an adsorption aptitude of 124.38 mg g^−1^, higher than the original MOF (107.70 mg g^−1^).^224^

The adsorption of Sulfate (SO_4_^2−^) ions from water was explored using stable NU-1000 (Zr-MOF). Within a contact time of just 1 minute, NU-1000 has shown 56 mg g^−1^ of phosphate uptake and this high rate was probably because NU-1000 has extensive 30 Å apertures, enabling the diffusion of SO_4_^2−^ ions easily throughout the structure. The 2130 m^2^ g^−1^ and 2045 m^2^ g^−1^ BET surface areas indicated before and after adsorption, and NU-1000 exhibits nearly identical N_2_ adsorption isotherms.^225^ Ba(BDC)-MOF prepared using barium metal ions and terephthalic acid ligands was a highly competent adsorbent showing 549.5 mg g^−1^ sulfate ion uptake and 99.43% removal efficiency from 200 ppm aqueous solution using 3.1 mg of adsorbent dose at 29 °C temperature.^226^ MOF-808 (Zr-BTC-MOF) and MIL-100 (Fe-BTC-MOF) were produced and their adsorption behavior towards *p*-cresyl sulfate was analyzed. MIL-100(Fe) has a higher surface area (1024–2200 m^2^ g^−1^) than that of MOF-808 (1710 m^2^ g^−1^). The adsorption capacities shown by MOF-808 and MIL-100(Fe) were 23.6 mol mg^−1^, and 68.6 mol mg^−1^ indicating that MIL-100(Fe) adsorbed *p*-cresyl sulfate three times more proficiently than MOF-808, surpassing 75% of Zr-MOFs reported earlier.^227^

### 4.9 MOFs as photocatalysts for the removal of oil and hydrocarbons

**Fig. 25:**
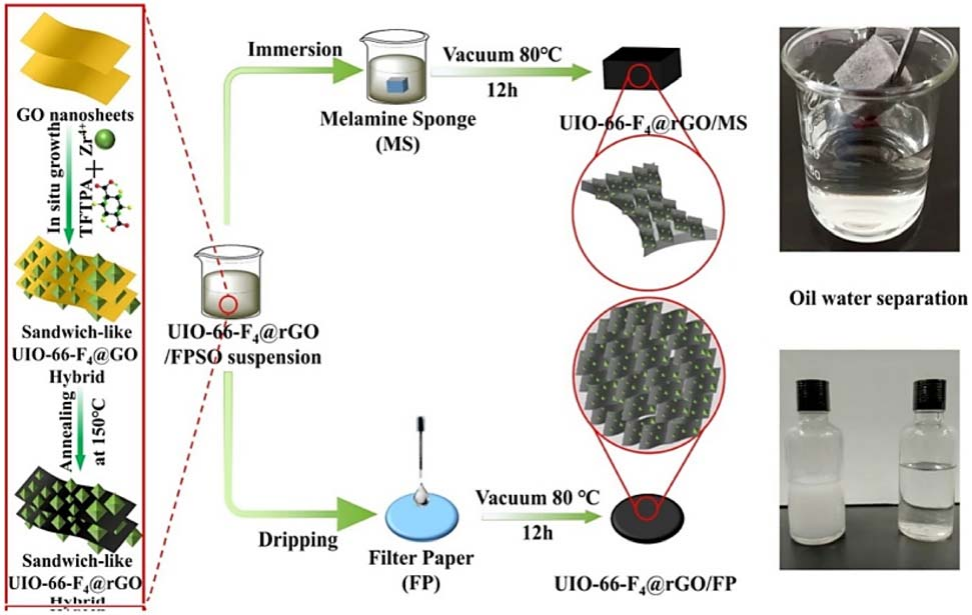
Oil–water separation using UIO-66-F4@rGO/MS as the nanocatalyst. Reproduced with permission from ref. 232. Copyright (2020) Elsevier.

UPC-21, a porous metal–organic framework with 1725.1 m^2^ g^−1^ surface area and strong hydrophobic characteristics, was synthesized. UPC-21's high hydrophobicity, lasting porosity and water stability enable the effective extraction of various organic pollutants from water sources. UPC-21 demonstrates outstanding separation efficiency with reported values of 99.4% for toluene/H_2_O, 99.0% for gasoline/H_2_O, 97.6% for crude oil/H_2_O, 99.2% for hexane/H_2_O and 99.2% for naphtha/H_2_O.^228^ MS-CMC-HPU-13, a composite material made up of porous, water-stable MOF and a melamine sponge (MS), exhibits strong oleophilic and hydrophobic properties. CMC-HPU-13 showed impressive absorption capacities for organic solvents and oils, along with reusability and environmental sustainability. The composite material exhibits a remarkable oil adsorption capacity of 13 000%, demonstrating its potential for effective oil spill cleanup.^229^ The *in situ* self-assembly of polyurethane foam with ZIF-8 MOF results in the formation of elastic modified polyurethane foam (MFPU). Leveraging the extensive surface area and high porosity of the FPU foam, along with the enhanced lipophilicity and hydrophobicity resulting from ZIF-8 growth, these combined advantages position the MFPU foam as an outstanding absorbent for oil/water separation. The synthesized foam demonstrates an absorption capacity 33 times more than that of its original weight.^230^ The highly stable, water repelling, micro-porous, flower-shaped ZIF-7 MOF nanoparticles were synthesized and analyzed for their adsorptive behavior towards oil/water separation. The coatings of ZIF-7 arrays exhibited a water contact angle of 154.7° and an exceptionally high flux range (5.4 × 10^4^–8.3 × 10^4^ L m^−2^ h^−1^). The MOF exhibited outstanding separation efficiencies exceeding 99.5% for *n*-hexane, toluene, soybean oil and petroleum ether, with a separation efficiency of 97% for dichloromethane.^231^ A 3D UIO-66-F4@rGO composite with high hydrophobic and oleophilic properties along with a large contact angle value of 169.3 ± 0.6° was found very effective in a number of oil/water separations (Fig. 25). UIO-66-F4@rGO/MS selectively absorbs oils at an adsorption capability of 26 to 61 g g^−1^ based on oil viscosity, enabling ongoing oil spill cleanup.^232^

**Fig. 26:**
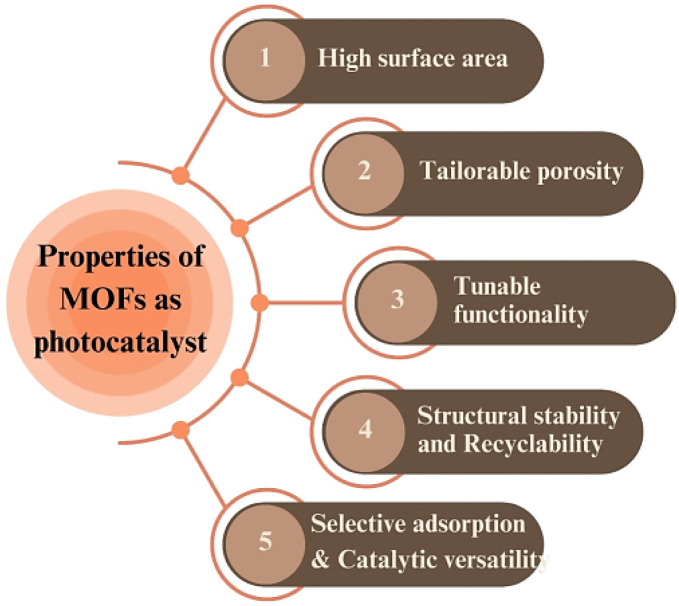
Properties of metal organic frameworks as photocatalysts.

The introduction of a Cu^2+^ paddlewheel-based MOF, [Cu_6_(C_22_SO_10_H_10_)_3_(DEF)_6_]·6(DEF), endowed a N_2_ adsorption-calculated BET area of 2410 m^2^ g^−1^. The Cu-MOF adsorption behavior towards C2-hydrocarbons (ethylene and acetylene) varies greatly with the temperature. At temperatures of 273 K and 298 K, the acetylene adsorption (241 and 160 mL g^−1^) was high compared to ethylene adsorption (215 and 160 mL g^−1^) by the CU-MOF at the standard temperature and pressure.^233^ Carbonized CD-MOF-2 (cyclodextrin-MOF-2) with 5 Å pore size selectively adsorbs normal butane and pentane than its structural isomers, with a calculated BET surface expanse of 799 m^2^ g^−1^. The adsorption equilibrium in the case of butane was achieved quickly just in 60 s while in the case of iso-butane, it contains more than 3500 s. At 1 bar pressure, the CD-MOF-2 uptakes for *n*-butane and *n*-pentane were 1.9 and 2 mmol g^−1^, while for iso-butane and iso-pentane, the uptakes were 1.43 and 0.7 mmol g^−1^.^234^ Two zeolitic imidazolate frameworks, namely ZIF-69 (SBET = 845.1 m^2^ g^−1^) and ZIF-8 (SBET = 1285 m^2^ g^−1^), were employed to study their adsorption behavior towards 2-methylpentane and *n*-hexane. Both MOFs showed high adsorption capacities for *n*-hexane as compared to 2-methylpentane. In the case of *n*-hexane, the adsorption capabilities of ZIF-8 and ZIF-69 were 0.51 and 0.34 g g^−1^, while for 2-methylpentane, the capacities were just 0.10 and 0.09 g g^−1^, which were due to the diffusional restrictions of 2-methylpentane. These results demonstrated that despite having an equal number of carbons in various hydrocarbons, the MOFs exhibited a selective uptake preference for particular hydrocarbons.^235^ A calcium sulfonyldibenzoate MOF having a distinctive crystal structure demonstrated shape-dependent adsorption of hydrocarbons, influencing the orientation of the hydrocarbon within the pores. Ca(sdb) MOF displayed an uptake of 0.9 heptane molecules per unit cell, yielding 0.64 mmol g^−1^ adsorption capacity and also for pentane, ethane, butane and propane, the adsorption capacities were 0.76, 1.42, 1.41 and 1.50 mmol g^−1^, respectively.^153^ MIL-101-Cr-SO_3_Ag and MIL-101-Cr-SO_3_H with specific surface areas (1374 and 1570 m^2^ g^−1^) were employed for ethane and ethylene adsorption. The Ag(i) ion introduction in MIL-101-Cr-SO_3_H results in higher ethylene adsorption uptake than original MIL-101-Cr-SO_3_H. The ethylene uptakes shown by MIL-101-Cr-SO_3_H at 296 K and 318 K were 42 cm^3^ g^−1^ and 37 cm^3^ g^−1^, which increased to 73 cm^3^ g^−1^ and 63 cm^3^ g^−1^, respectively by using MIL-101-Cr-SO_3_Ag. Furthermore, there is no significant difference between ethane adsorption of two MOFs, indicating that there is no pronounced effect of Ag^1+^ ions on the adsorption of ethane.^236^ Fluoride is often added to drinking water for its crucial role in promoting dental health and treating osteoporosis. High fluoride levels (>1.5 mg L^−1^) in water can harm human health, leading to disorders such as Alzheimer's disease, fluorosis, infertility, DNA damage, and kidney failure.^237^ Therefore, for the removal of fluoride ions, a number of MOFs have been synthesized. ZIF-8 nanoparticles (200–300 nm) were strong enough to remove 92% fluoride ions from water under optimized conditions and after three consecutive cycles, the removal rate is still high, *i.e.* 87%. The fluoride ion uptake was 90 mg g^−1^ during this period.^238^ MOF-801, a fumarate-derived MOF, having 755 m^2^ g^−1^ surface area was utilized to selectively eliminate fluoride from brick tea infusion. The adsorption behavior of MOF-801 was temperature dependent, *i.e.* at 25 °C, the adsorption capability was 32.13 mg g^−1^ and at high temperature (100 °C), the adsorption capacity increases to 166.11 mg g^−1^.^239^ Zr-MOFs, both as an adsorbent and a membrane, possessing an exterior area of 740.28 m^2^ g^−1^, were employed for fluoride ion adsorption. At a pH of 7.0 and an adsorbate concentration of 200 ppm, the Zr-MOF adsorbent demonstrated a *Q*_max_ value of 102 mg g^−1^. The fluoride ion removal rate shown by 20 μm-thick Zr-MOF's membranes decreases with the increase in adsorbate concentration, *i.e.* at 5, 8 and 10 ppm concentrations, the fluoride removal rates were 5510, 5173, and 4664 L m^−2^, respectively,^240^ as shown in Table 2. Hydrothermal synthesis was employed to synthesize MIL-96(RM) using metal ions extracted from red mud (RM) for the elimination of fluoride from water. The specific surface area of MIL-96(RM) calculated using BET analysis was 168.26 m^2^ g^−1^. The ion exchange was the probable mechanism for fluoride adsorption, and 82.645 mg g^−1^ adsorption capacity was recorded at 20 °C.^242^ Two lanthanum-based MOFs (La-BDC and La-ABDC) were hydrothermally synthesized using two different ligands [terephthalic acid(BDC) and aminobnzene-1,4 dicarboxylic acid (ABDC)]. According to BET analysis, the La-ABDC-MOF has a higher surface area (10.15 m^2^ g^−1^) than that of La-BDC-MOF (5.25 m^2^ g^−1^) due to the presence of active amine functionalities. The fluoride uptake of both MOFs was close to 4950 mg kg^−1^ for La-ABDC-MOF and 4920 mg kg^−1^ for La-BDC-MOF.^241^ The properties of MOFs as photocatalysts are shown in Fig. 26.

**Table 2:** Synthetic parameters of MOFs and their adsorption capacity for pollutants

MOFs	Synthesis method	Surface area (m^2^ g^−1^)	Pollutant	Adsorption capacity of pollutants (mg g^−1^)	Reference
[Ni_2_F_2_(4,4′-bipy)_2_(H_2_O)_2_](VO_3_)_2_·8H_2_O	Ultrasonication	—	Pb(ii)	2400.7	110
Fe_3_O_4_@UiO-66-NH_2_	Co-precipitation	287	Cd(ii)	714	116
*n*Fe_3_O_4_@MIL-88A (Fe)	Microwave assisted extraction	62.21	Cd(ii)	755.8	115
Zr-TDA	Hydrothermal	74.37	Hg(ii)	605.5	120
UiO-66-36-TFA	Hydrothermal	1690	As(v)	200	127
Cd (tipo)(HCOO)(H_2_O)]·NO_3_·DMF	Solvothermal	—	Cr(vi)	228	131
Fe_3_O_4_-ethylene diamine/MIL-101(Fe)	Solvothermal	300	Cr(iii)	173	130
NH_2_-Cu-MOF	Solvothermal	840	Bacterial endotoxin	2500	195
MOF-545/PCN-222	Solvothermal	2336	Methylene blue	906	134
MIL-53(Al)	Solvothermal	610.5	Rhodamine B	1547	141
3D Zn-MOF	Solvothermal	102.36	Congo Red	355.16	147
MIL-101-Cr MOF	Solvothermal	2410	Reactive Black 5	377–397	151
Zn-MOF	One-pot synthesis	1820.7	Malachite green	953.14	155
Al-MOF/SA-CS composite	Multi-step synthesis	687.54	Bisphenol A	136.9	163
NH_2_-MIL-88B	One-pot reflux	414	2,4,6-Trinitrophenol (TNP)	163.66	164
Fe_3_O_4_@ZIF-8	Ultrasonication	942	Toluene	133	168
Fe_3_O_4_@ZIF-8	Ultrasonication	942	Benzene	148	168
ZIF-8	Hydrothermal and steam assisted	—	Ethion, prothiofos	279.3	172
				366.7	
ZIF-67	Hydrothermal and steam assisted	—	Ethion, prothiofos	210.8	172
				261.1	
CaFu MOF	Solvothermal	2308.03	Imidacloprid pesticide	467.2	114
Sn-MOF	One-pot synthesis	897.6	Diazinon pesticide	587.3	173
[EMIM][In(ABDC)_2_]·DEF·H_2_O	Solvothermal	307	Carbon dioxide	81.3	176
PAF-302 MOF	—	5600	Sulphur dioxide	50.69	183
ELM-12	—	706	Sulphur dioxide	61.2	185
UiO-66-1.0HAc	Hydrothermal	1450.1	Benzene and toluene	367.13, 410.21	186
UTSA-76	Solvothermal	—	U(vi)	91.31	207
UiO-66-(COOH)_2_	Hydrothermal	250	Th(iv)	360	209
CAU-1 NH_2_	Solvothermal	550	Th	404	211
MOF-808-SO_3_H	Hydrothermal	683.8	Ba^2+^	152.0	216
Zr-BDC-NH_2_-SO_4_	One-pot reflux	374	Ba^2+^	Of 181.8	215
LTA-MOF	Solvothermal	129	NO^3−^	50.09	218
Fe-MIL-88B	Solvothermal	165.45	NO^3−^	92.59	221
Fe/Al (NO_3_^−^) MOF	Solvothermal	945	PO_4_^3−^	130	223
MIL-101(Fe) MOF	Solvothermal	2350	PO_4_^3−^	107.70	224
NH_2_-MIL-101(Fe)	Solvothermal	2736	PO_4_^3−^	124.38	224
LTA-MOF	Solvothermal	129	PO_4_^3−^	63.01	218
NU-1000	—	2130	SO_4_^2−^	56	225
MIL-100(Fe-BTC-MOF)	Solvothermal	1024–2200	*p*-Cresyl sulfate	68.6	227
[Cu_6_(C_22_SO_10_H_10_)_3_(DEF)_6_]·6(DEF)	—	2410	Acetylene	241	233
			Ethylene	215	
ZIF-69	Simple stirring	845.1	*n*-Hexane	0.51	235
MIL-101-Cr-SO_3_Ag	Hydrothermal	1374	Ethylene	73	236
MOF-801	Solvothermal	755	Fluoride	166.11	239
Zr-MOF	Solvothermal	740.28	Fluoride	102	240
La-BDC-MOF	Hydrothermal	5.25	Fluoride	4920	241
La-ABDC-MOF	Hydrothermal	10.15	Fluoride	4950	241

## 5 Recent advances in MOFs as catalysts

### 5.1 MOFs for water splitting

In recent years, metal–organic frameworks (MOFs) have gained interest in water splitting because of their high porosity and design flexibility. Cheap and efficient methods of H_2_ generation are processes such as water splitting that break water into hydrogen and oxygen. Cathodic polarization treatment (CPT) after zeolitic imidazolate framework-8 (ZIF-8) has demonstrates high HER performance in 0.5 M H_2_SO_4_ electrolyte and exchange current density of 0.063 mA cm^−2^.^243^ Co-MOF-74 and MoS_*x*_/Co-MOF-74 were prepared by solvothermal synthesis using rod-like Co-MOF-74. The mixed metal–organic framework Co-MOF-74 demonstrated an efficient way of enhancing the catalytic HER performance using (NH_4_)_2_MoS_4_ as the precursor material. Mesoporous rod-like Co-MOF-74 was synthesized by solvothermal synthesis and had a surface area of 335.4 m^2^ g^−1^.^244^ A hybrid catalyst of Mo_2_C and Zr-based metal–organic framework (UiO-66) was synthesized through the solvothermal process. Particularly, the optimized Mo_2_C/UiO-66 hybrid, termed MCU-2 with 50 : 50 wt% of both components showed the best catalytic activity regarding the HER/OER. It could provide a small over potential of 174.1 mV to achieve a current density of 10 mA cm^−2^ and a Tafel plot slope of 147 mV dec^−1^ for HERs.^245^ For similar reasons, metal–organic frameworks prove to be a promising material for water splitting, but there are some issues such as instability in water environment, low band gap and poor efficiency of charge transfer in MOFs. They frequently require co-catalysts for HERs and OERs, and the fabrication of new materials is difficult and expensive. Future work concerns the improvement of MOF stability, light absorption and charge transfer processes. It has been observed that visible light efficiency is improved with the help of metal doping and conjugated linkers and the water stability of MOFs. The development of large format hydrogen synthesis using sustainable approach and the application of MOFs in combination with solar systems are important in hydrogen production on an industrial scale.

### 5.2 MOFs for CO_2_ reduction

Metal–organic frameworks (MOFs) show potential for capturing CO_2_ and converting it into useful chemicals and fuels. Due to their high surface area, the flexibility of pore size and surface, as well as the possibility of the integration of catalytic sites, they are highly efficient for use in CO_2_ reduction reactions (CO_2_RR). Co-PNN or Mn-PNN PCN-222 show high performance for photocatalytic CO_2_ reduction due to its enhanced activity of transition metal ions such as Co and Mn ions. The porphyrin ring facilitates improved light absorption to cater the process of transformation of CO_2_ into CO and formic acid when irradiated under visible light.^246^ Ti^4+^ ions in the MOF-74 structure had been incorporated *via* a one-pot hydrothermal synthesis process. The obtained Ti^4+^-doped MOF-74 photocatalysts were found to possess enhanced performance in the reduction of CO_2_ into CO. The doping of Ti^4+^ ions generate energy bands beneath the conduction band minimum of MOF-74, which widened the visible light response range and made the photocatalysts work under additional light spectrum for the catalytic reactions.^247^ UiO-66(Zr/Ce) successfully introduced nanosheets featuring on g-C_3_N_4_ [g-C_3_N_4_/UiO-66(Zr/Ce)] using single-atom metal precursors of zirconium (Zr) and cerium (Ce) through *in situ* synthesis. g-C_3_N_4_/UiO-66(Zr/Ce) does not require any additional sacrificial agent and it exhibits good CO_2_ reduction performance for CH_3_OH (54.71 μmol h^−1^ g^−1^) and C_2_H_5_OH (38.10 μmol h^−1^ g^−1^).^248^ The Cu^0^/Cu^+^ interface in Cu-MOF74/Cu_2_O-350 improves the adsorption of reactive intermediates providing more active sites available for CO_2_ reduction. The exhibited material composite depicts desirable CO_2_ reduction characteristics. When the potential was at −1.3 V *vs.* RHE, the theoretical current density for C_2_H_4_ production was up to 32.48% FE, which was much higher than that of Cu_2_O-350 (9.25% FE) and Cu-MOF74-350 (15.52% FE).^249^ MOFs also exhibited CO_2_ reduction owing to a wide surface area with adjustable porosity and adjustable active sites, which facilitate CO_2_ adsorption and catalytic activity. This makes it easy to link them with metal catalysts or other light absorbing parts raising the efficiency of CO_2_ to fuel conversion rate. New directions include the synthesis of even more stable and effective MOFs, collection of efficient charge separation, and combining them with other materials. Low-cost, environmentally benign synthesis processes and integration of the MOFs with renewable energy resources are likely to be critical in the further enhancement of CO_2_ conversion for efficient synthesis of fuels.

### 5.3 Photo-catalysis

Metal–organic frameworks (MOFs) have gained much interest in the photocatalytic field due to the photophysical and electronic properties presented by MOFs, which make them suitable for promoting photocatalytic processes such as degradation of pollutants, water splitting, and CO_2_ reduction under light. Hydrogen generation through photocatalytic activity using stable MOFs, especially the titanium-based MOFs (Ti-MOFs), is one of the probable solutions for energy problems. Facilitating those structural characteristics, NH_2_-ZSTU-2 exhibited a stable hydrogen production rate under visible light exposure, averaging at 431.45 μmol g^−1^ h^−1^ with triethanolamine and Pt as terminal electron donors and co-catalysts, respectively, almost impressively 2.5 times higher than ZSTU-2.^250^ The synthesis and characterization of two new coordination MOFs, namely Monometallic Co-MOF(DABCO) and bimetallic NiCo-MOF(DABCO) with the chelating agent DABCO, and the determination of their various physicochemical properties were reported. Based on RSM results, the predictability of the tested conditions in CP degradation efficiency was considered to be satisfactory and was further validated using ANOVA (*p* < 0.05) and obtained a *R*^2^ value of 0.99. NiCo-MOF(DABCO) exhibited a cefoperazone (CP) removal efficiency of 92.34%.^251^ A capsule-like bimetallic porphyrin-based MOF called PCN-222(Ni/Hf) has been synthesized by a simple hydrothermal process. The adsorption/photocatalytic efficiencies were investigated using four representative dye molecules, namely RhB, BV14, CV, and AB210, and the Ni/Hf bimetallic PCN-222 showed improved overall removal efficiency relative to monometallic Hf PCN-222.^252^ Photocatalysis has benefits like using energy from the sun to fuel the reactions, embracing sustainable solutions like hydrogen production and pollution remediation. The efficiency for this photocatalytic process is derived from the sustainable sources of light and the flexibility in the design of the catalyst. The future directions involve increasing the photocatalytic activity under visible light, prolonging their useful life, and incorporating them into solar-driven systems in environmental and energy applications on an industrial scale.

## 6 Current challenges and future perspectives

Growing environmental pollution has become a global problem due to industrial activities, urban development, and a lack of waste disposal policies. The old techniques of environmental restitution are likely to have failed to make the issue just as complex and as large as the existing pollution problems. Historically, the use of MOFs for cleaning up pollutants has been a game-changer for environmental treatment efforts across the world. Although a lot of progress has been made in this field, a few barriers and challenges remain, while there are tremendous opportunities for future acceleration in this sphere. One of the big difficulties in using MOFs as an environmental remediating technology is the lack of widely accessible synthesis methods. The existing methods do result in the production of low-yield MOFs, which effectively hinders their application in large-scale remediation. One of the catalysts that can speed up the large-scale manufacturing of MOFs while guaranteeing the integrity of their structure and catalytic activity is promising for synthesis techniques. For instance, a large number of MOFs manifest exceptional catalytic activity in the laboratory under controlled conditions but demonstrate a negative correlation between their observed efficiency and real-world situations such as atmospheric temperature fluctuations, humidity, and the presence of chemical impurities. To make it possible for MOFs to perform at their best under harsh environmental conditions that limit their long-term performance, stability and resilience properties are quite important. Although MOFs have a greater surface area and pore size, the separation of the comprehensive mixtures is the main issue since it is very difficult to selectively adsorb the necessary pollutants. The targeted modification of MOFs to remove specific pollutants while reducing the interference posed by co-existing contaminants is a fundamental requirement for efficient pollutant removal. This would be done by the use of MOFs that are made from expensive precursors and intricate fabrication processes; the costs of their production would increase. Economically competitive industrial-scale cleaning applications are made possible by cost-effective synthesis routes and scalable production technology development. The MOF regulatory approval process for novel ecological cleaning technologies was slow and difficult due to the high requirements. Setting up clear requirements and standards for the evaluation of the safety, efficacy, and adaptability of MOFs will pave the way for their approval in remediation projects. Future research will concentrate on engineering representational MOFs with customized shapes and capacities that are effective at cleaning for those pollution remediation processes. The use of computational modelling and high-throughput synthetic techniques in the structure approach leads to MOFs with increased selectivity, stability, and catalytic activity. The merging of different functionalities inside the MOF compounds, for example, catalysis, sensing, and adsorption, would apparently create the most significant multifunctional environmental remediation systems. This type of MOF, capable of synergistic effects as well as better functionality, may lead to very efficient and diverse solutions for pollutant removal. Intrusive remedial applications using MOFs offer the opportunity for direct treatment of contaminated sites without as much landscape removal or transportation of dirty stuff. In-place MOF-based systems, which will be able to clean up the pollutants will minimize disturbance of the environment and reduce the costs of cleaning up. The emulation of natural systems and bio-inspired MOFs with biomimetic features and functions are being implemented now for environmental remediation. Imitating the natural processes of enzymatic catalysis or molecular recognition in MOFs creates a framework for eliminating pollutants with enhanced efficiency and specificity. The inclusion of sensors into the framework of MOFs helps to monitor these pollutants in real time and take the necessary remedial measures quickly, depending on their severity. MOF-based smart sensors that can detect and measure pollutants accurately and selectively with high sensitivity will definitively change the game in terms of environmental surveillance and remediation. In the end, although issues do arise during the popularization of MOFs concerning pollution remediation, continuous efforts in research and technological development bring new hopes for a ground-breaking change in environmental cleanup practices. If present-day critical points are considered, as well as opportunities for the future, MOFs will most probably become irreplaceable weapons against environmental pollution and protect our environment from the next generation of people.

## 7 Conclusion

This review has considered the evolution of MOFs and their remarkable impacts on counteracting environmental pollution issues. However, the functionality of MOF surfaces with catalytically active sites is another factor that increases catalytic activity and selectivity for pollutant degradation reactions. One of the utmost features of MOFs is their ability to respond to different environmental conditions and different pollutants by being flexible. Furthermore, multifunctional catalysts that effectively remove many pollutants in a single operation can be designed by leveraging the flexibility of MOF frameworks. These kinds of treatments are therefore ideal for environments with a variety of pollutants, including those found in the heavy metal industries. Furthermore, these strategies-such as post-synthesis modification, bottom-up assembly, and sophisticated characterization are presently applicable and have the potential to play bigger roles in the treatment of pollutants. These advances enable the making of MOFs with higher stability, reusability, and effectiveness, which further facilitates the development of scalable and economical environmental cleanup methods. In order to achieve that specific goal, the remaining challenges that stand in the way of successfully applying MOFs for pollution remediation should also be addressed. The questions associated with MOF stability and synthesis process scalability, as well as the lack of integrated systems for MOF regeneration and reuse, belong to these matters. More importantly, collaboration among materials scientists, environmental engineers, and policymakers is paramount to speeding up the transfer of MOF-based technology from the laboratory to the field.

## Data availability

The data presented in this review are available from authors upon request.

## Author contributions

Umme Farwa and Zeshan Ali Sandhu designed the project and wrote the original draft. Azwa Kiran, Muhammad Fiaz and Adnan Malik collected the data. Muhammad Asam Raza supervised the whole project. Sufyan Ashraf and Hamza Gulzaraib carried out the graphical work. Abdullah G. Al-Sehemi helped in finally polishing this work.

## Conflicts of interest

All authors declare that they have no conflict of interest.
